# Ultra‐Confinement of Polaritons in Single Atomic Layer Ag Photonic Quantum Dots

**DOI:** 10.1002/adma.202521015

**Published:** 2026-05-26

**Authors:** Xinyi Li, Tetyana Ignatova, Chengye Dong, Krishnan Mekkanamkulam Ananthanarayanan, Rinu Abraham Maniyara, Arpit Jain, Furkan Turker, Vinay Kammarchedu, Aida Ebrahimi, Joshua A. Robinson, Slava V. Rotkin

**Affiliations:** ^1^ Department of Engineering Science and Mechanics The Pennsylvania State University University Park Pennsylvania USA; ^2^ Department of Nanoscience Joint School of Nanoscience and Nanoengineering The University of North Carolina at Greensboro Greensboro North Carolina USA; ^3^ Two‐Dimensional Crystal Consortium The Pennsylvania State University University Park Pennsylvania USA; ^4^ Department of Materials Science and Engineering The Pennsylvania State University University Park Pennsylvania USA; ^5^ Department of Electrical Engineering The Pennsylvania State University University Park Pennsylvania USA; ^6^ Department of Biomedical Engineering The Pennsylvania State University University Park Pennsylvania USA; ^7^ Department of Chemistry The Pennsylvania State University University Park Pennsylvania USA; ^8^ Department of Physics The Pennsylvania State University University Park Pennsylvania USA

**Keywords:** 2D metals, epitaxial graphene, hyperspectral maps, nanostructures, polaritons, sSNOM, sub‐wavelength resolution

## Abstract

Light scattering by two‐dimensional (2D) van der Waals heterostructures (vdWHs) is immense, especially given their infinitesimal volume, thus enabling strong light‐matter interactions. Surface 2D polariton waves manifest through a large concentration of electromagnetic field in the vertical direction, normal to their propagation. By confining vdWH materials into 2D photonic shapes, one can manipulate and compress light in lateral directions. Scattering‐type scanning near‐field optical microscopy is a perfect tool for direct imaging of the propagating polaritons and studying the properties of confined polaritons in nanostructures. Though, thus far, the quantitative analysis, such as the wavelength extraction, has been challenged for confined polaritons by incapability of mapping of the wave period on a sub‐wavelength scale and the difficulty of identifying an adequate substrate's “background” to subtract. Here, an analytical approach is developed to reveal the local propagation constant of confined polaritons under the above‐mentioned constraints and map it with sub‐wavelength resolution. Applied to the analysis of the SiC/2D‐Ag/EG (epitaxial graphene) photonic nanostructures, the technique uncovered that the polaritons are highly confined in both vertical (∼*λ*/50) and lateral directions (∼*λ*/40) by 2D metal.

## Graphical abstract

Strong confinement of surface phonon polaritons of silicon carbide was achieved in photonic quantum dots fabricated from van der Waals heterostructure of epitaxial graphene over single‐atomic layer silver. Scattering‐type scanning near‐field optical microscopy combined with an analytical approach, developed to reveal local propagation constants with the sub‐wavelength resolution, revealed the polariton confinement ∼*λ*50 in vertical and ∼*λ*40 in lateral direction.

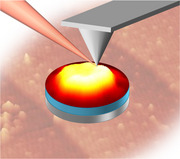

## 1 Introduction

Light squeezing phenomenon in surface polariton and two‐dimensional polariton (SP/2DP) waves, which manifests with the wavelength being much smaller than that of the bare light in vacuum, enables strong light‐matter interactions. Since the photon energy density (and the light intensity) is inversely proportional to the wavelength, the light squeezing allows for achieving large fields, leads to non‐linear physics, and opens up possibilities for a number of fundamental studies in quantum optics as well as some novel applications. While the large energy density for propagating/free SPs/2DPs is due to the dielectric contrast at the interface, an additional light confinement can be reached through localization of the polaritons in plasmonic shapes, such as: photonic dots/metaatoms, metasurfaces, waveguides, nanoantennas, and resonators, typically made of low‐dissipation metals [1–3]. The range of materials used to fabricate plasmonic structures has recently been extended to two‐dimensional materials (2DMs). Highly confined polaritons in nanostructures based on 2DMs and their van der Waals heterostructures (vdWHs) have gained increasing interest in recent years [4, 5, 6, 7, 8, 9, 10, 11, 12, 13, 14, 15, 16, 17] with their strong light‐matter interactions as well as a capability to manipulate the latter at the scales down to a single atomic layer [18]. Atomically thin 2DMs possess unique properties compared to their 3D counterparts [19] and, even without patterning, support various surface polaritons [20, 21, 22]. When integrated into vdWHs, they enable polariton confinement [20, 23, 24, 25] which opens up exciting possibilities for developing next‐generation optoelectronic [23], photonic [26, 27], and sensing technologies [28, 29].

Here, we demonstrate ultra‐high confinement of mid‐infrared (MIR) surface‐phonon polaritons formed in the vicinity of a Reststrahlen band of polar SiC material. SiC samples are used as a substrate for confined heterostructure epitaxy synthesis of 2D atomically thin layers of various metals and other materials, encapsulated by epitaxial graphene (EG), including but not limited to In, Ga, and Sb [30, 31, 32, 33]. In this work, a 2D‐Ag monolayer is the material of choice due to its strong non‐linear optical response [34, 35]. These samples support an evanescent SP confined along *z*, a normal direction, which is a composite SP mode of SiC/2D‐Ag/EG vdWH. While it should propagate freely in the *x*–*y* plane in the as‐synthesized material, a lateral confinement is achieved by fabricating a photonic dot of the 2D‐Ag/EG.

To evaluate the plasmonic performance of the vdWH photonic dots, we use scattering Scanning Near‐field Optical Microscopy (sSNOM) [20] to experimentally determine both the SP/2DP wavelength, *λ_SP_*, and the confinement factor (CF). The CF is related to the leak of the electromagnetic field outside of the photonic dot area, which is defined by the magnitude of SP at the two sides of the boundary of the plasmonic nanostructure. The method developed here allows us to explicitly evaluate the spill‐out of the photonic wavefunction beyond the physical dimension of the confinement shape. We will show that this spill‐out is negligible in SiC/2D‐Ag/EG vdWH photonic dots of sub‐micron size, being substantially smaller than the SP wavelength.

sSNOM is an ideal tool to map free SP waves and, thus, directly measure their dispersion relation [20], that is, the wavevector *k_SP_* (and dissipation length as an imaginary component of the complex wavevector) as a function of SP frequency (sSNOM excitation frequency), as well as to detect nanoscale optical non‐uniformities that could affect photonic properties [29]. However, a common approach [36] – hyperspectral mapping of the periodicity of standing wave fringes vs. the excitation wavelength, *λ_o_* – is limited to plasmonic systems larger than *λ_SP_*. Since the size of the photonic dots in our samples is smaller than *λ_SP_*, the sSNOM cannot visualize the full wave period, in contrast to ref. [11], e.g., and the standard analysis fails to determine *k_SP_* and quantify the ***lateral*** light squeezing.

Furthermore, the wave periodic patterns in the sSNOM signal are combined with a background that has a negligible spatial dependence for uniform materials. However, this background varies substantially across the edge of the photonic structure, prohibiting the determination of actual SP wave amplitude difference from the sSNOM map and, thus, obscuring the definition of both the wave period and the CF. This is a notorious problem of proper referencing of the sSNOM signal, i.e., subtracting an unknown background signal.

To overcome this challenge, we developed an analytical approach for sSNOM hyperspectral mapping to reveal the local value of SP/2DP propagation constant and map it with deep‐sub‐wavelength resolution, only limited by the sSNOM noise level. This new method is based on the eikonal [37] representation of the SP wave. By tracing the phase increment in the complex space of the demodulated near‐field signal and analyzing the trajectory of wave phasors (in Argand diagram of original sSNOM data), we determine the propagation constant of confined surface polaritons for 2D‐Ag/EG plasmonic quantum dots with sub‐wavelength size, lithographically fabricated on SiC substrate. The method will be demonstrated to simultaneously mitigate the referencing/normalization problem by subtracting the spatially independent background and, thus, potentially enable optical nano‐spectroscopy of polaritonic materials by taking a hyperspectral sequence for SP/2DP maps.

## 2 Results and Discussion

**FIGURE 1:**
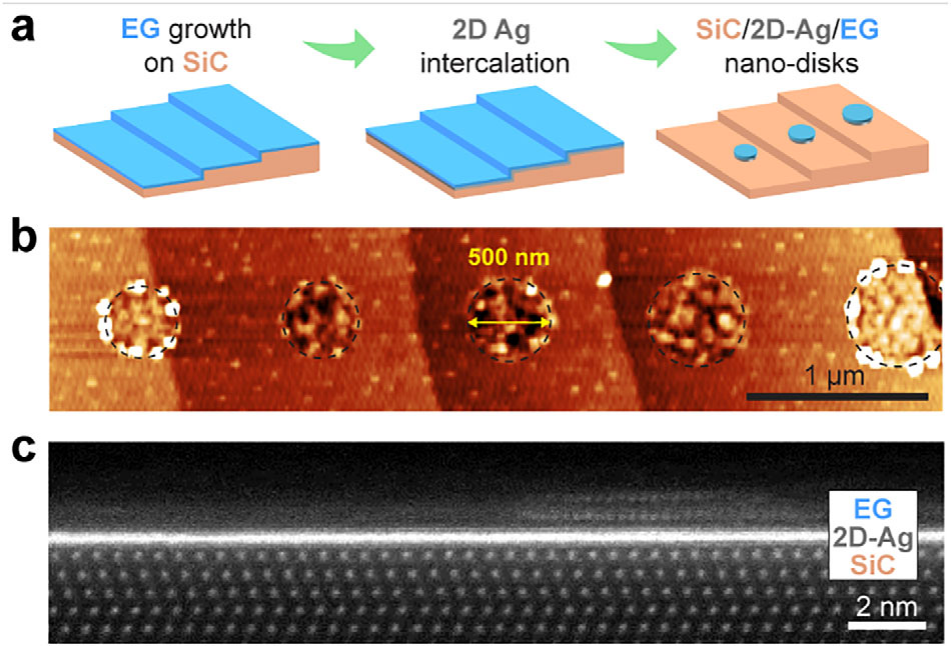
Material composition of 2D‐Ag/EG plasmonic structures. (a) Illustration of the fabrication process for 2D‐Ag/EG photonic quantum dots. (b) AFM topography of a series of disk‐shaped dots. Scale bar is 1 µm. (c) Representative STEM image of as‐synthesized SiC/2D‐Ag/EG material. Scale bar is 2 nm.

Cross‐sectional scanning transmission electron microscopy (STEM) has been performed to determine morphology and material composition in the original SiC/2D‐Ag/EG sample, before lithography. In Figure 1c, a typical cross‐section of the vdWH sample shows that Ag forms a single atomic layer (of brightest contrast) between the SiC substrate and EG, making up to 2 encapsulating graphene layers on the top. The photonic quantum dot structures were fabricated in vdWH material by e‐beam lithography (see schematics of the process in Figure 1a) with a range of sizes, all smaller than 1 micrometer in diameter, as shown in the Atomic Force Microscopy (AFM) topography image in Figure 1b. Black dashed lines indicate the boundaries of nano‐disks as a guide for the eye. Surface morphology of the samples shows “snake‐like” features [30] of ∼0.7 nm height that are consistent with an incomplete irregular second layer of EG.

**FIGURE 2:**
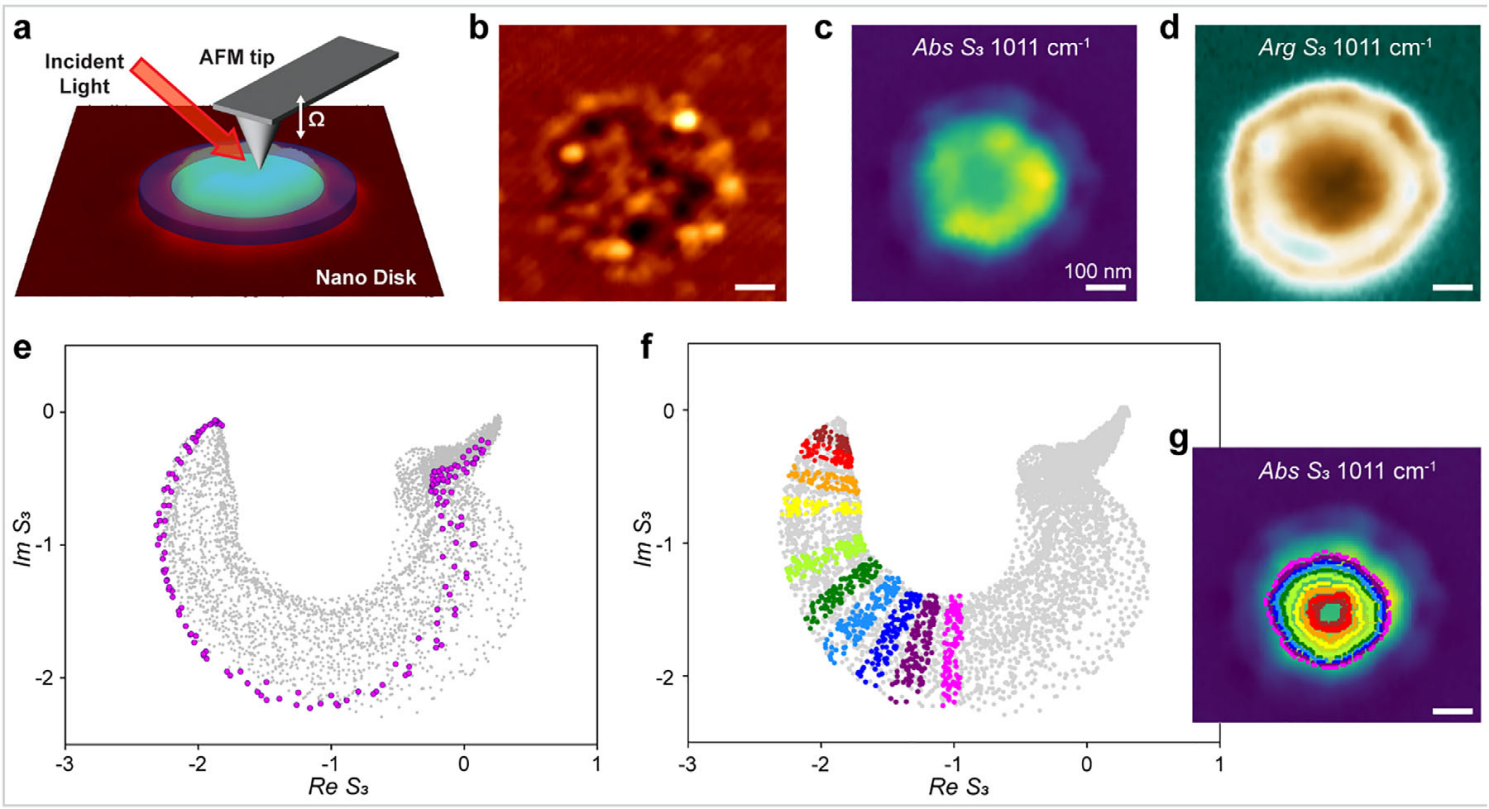
Surface polariton waves revealed by eikonal analysis. (a) Schematics of sSNOM imaging of the SiC/2D‐Ag/EG nano‐disks. (b) The AFM topography image; the raw maps of (c) third demodulated harmonic sSNOM optical amplitude, Abs(S_3_), and (d) optical phase, Arg(S_3_). (e,f) Argand plot: cross‐correlation between Re(S_3_) and Im(S_3_) signals (purple dots in (e) are taken along a fixed angular direction in real space image). (f,g) Correlation between the selected cluster points in Argand space with the same in the spatial map of Abs(S_3_). Scale bars are 100 nm.

Hyperspectral sSNOM mapping of the region of interest – the central nano‐disk of ∼500 nm diameter, marked with a yellow arrow in the AFM image in Figure 1b and shown in a magnified AFM image in Figure 2b – was performed in the MIR range: 963–1040 cm^−1^. Notably, this region covers the SiC LO phonon band. Figure 2 shows representative single‐excitation‐frequency images of the third‐harmonic demodulated sSNOM raw signal: (c) optical amplitude, Abs(S_3_), and (d) optical phase, Arg(S_3_), compared to disk geometry from the AFM topography image in panel (b). While the fact of the SP confinement is seen by the brightness/contrast of the map – a higher (raw) optical amplitude signal inside the nano‐disk compared to the outside region (substrate) – as well as via the phase contrast, the degree of this ***lateral*** confinement cannot be quantified from the Abs(S_3_) signal because it also contains an unknown background step at the edge, besides the SP wave amplitude contrast. We propose an approach to split the contributions from the SP wave and from the materials’ dielectric contrast by fitting the SP eikonal wave. Additionally, both the phase and amplitude maps allow one to trace a narrow belt around the disk edge where the signal is lower than in the disk center, though still much higher than that of the substrate (see Figure S1). This belt is likely a silver oxide material formed due to self‐limited oxidation of the edge of 2D‐Ag, which will be confirmed by SP eikonal analysis below. Such additional lateral heterostructures are often formed in 2D vdWH systems, which makes the standard SP analysis even harder. Markedly, there is no clear peak‐to‐trough pattern formed within the nano‐disk due to its sub‐diffraction size with respect to the SP wavelength, when the belt is excluded from the wave period analysis.

Figure 2e shows the Argand plot, i.e., the correlation plot between imaginary and real parts of the complex‐valued S_3_ sSNOM signal, taken from the whole map (gray points) at the excitation wavelength of 1011 cm^−1^. In this Argand plot, a series of arc shapes is clearly seen. These arcs in the complex plane correspond to eikonal waves of a nearly constant amplitude and variable phase $:\;M\;\;e^{i\phi }=M\;e^{iks}$ where the phase evolution ϕ=ks is related to *k*, the SP wavevector, and *s*, the distance along the propagation direction. For example, the trace of an evolution of the phasor (complex vector) of the S_3_ signal along one of the radial directions from the center of the plasmonic dot toward the substrate region is shown as a series of purple points. Two adjacent arc segments of the phasor trajectory are seen, of a larger and small radius, that correspond to two SP eikonal waves in the 2D‐Ag dot itself and in the oxide belt next to it, with different background (reference) signal, magnitude, velocity, and phase of SP modes (that correspond to arc center, radius, tangential derivative, and start phase, respectively). We emphasize that the true eikonal wave phase velocity computed along the phasor trajectory equals the local SP propagation constant *k*. Notably, this quantity can be computed, in the ideal case of negligible noise, with the resolution of 2× pixel size of the raw sSNOM map, which is far beyond any other existing analytical near‐field method.

To further prove that the phasor arcs in the Argand plane correspond to different SP trajectories, we use the fact that in this particular map, all arcs are nearly concentric (have a common center). Neglecting for a moment the difference in actual center position, i.e., temporarily postulating a constant background signal (a single center for all data points), we can define narrow regions along a fixed radial direction – “spokes” of different colors in Figure 2f. Such a set of points with nearly the same SP phase should correspond to the eikonal wavefront. Indeed, Figure 2g shows that these points correspond to continuous circular curves in the real‐space map, marked with the same color as the corresponding spoke. The radial shape for the confined SP eigenmode is typical for the axial symmetry of the nano‐disk. We emphasize that the shape of the wavefronts, unlike the raw amplitude (or phase) signal, is robust to local defects and other perturbations due to the disk morphology, seen in the AFM image in panel (b). Additional evidence for the negligible influence of the surface morphology on the sSNOM signal and, therefore, the eikonal analysis is provided in Section S7.

While the radial spoke of constant phase in Argand space corresponds to the wavefront in real space, the displacement along the arc trajectory in Argand space corresponds to the evolution of the eikonal phase, i.e., the wave propagation normal to the wavefront. By tracing the segments of an individual arc picked in the Argand space in Figure S3a, the SP wave evolution in the radial direction is seen in Figure S3b. Note that the arc segments of approximately the same angular size (arc length) do not appear equally spaced in the map in panel (b). Indeed, this reflects on the change of the phase velocity along the radial direction: equidistant regions in real space in Figure S3d result in the variable incremental arc length of the segments of the same color in Figure S3c.

**FIGURE 3:**
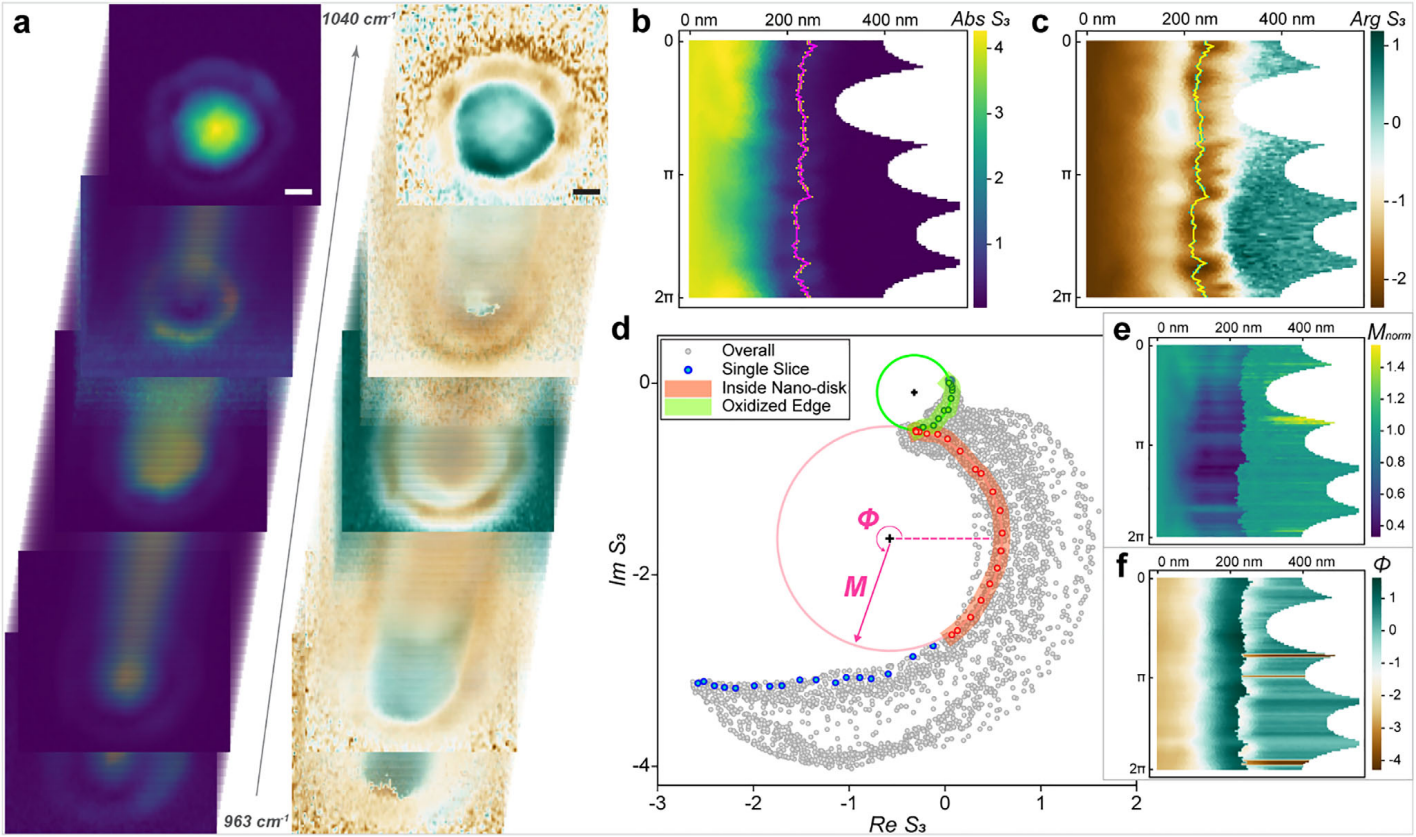
Spectral analysis of eikonal wave model. (a) Sketch of the hyperspectral sSNOM mapping using excitation wavelength 963–1040 cm^−1^. Scale bars are 100 nm. (b,c) Representative sSNOM Abs(S_3_) and Arg(S_3_) maps at excitation wavelength 994 cm^−1^, after polar coordinate transformation. (d) Argand space representation of data from (b,c); the datapoints selected along a single radial direction are fitted to two eikonal curves (red and green scatter dots and arcs, the centers are marked by black crosses). The coordinates of adjacent points between two eikonal waves are shown in panels (b,c) as magenta/yellow dots. (e,f) Fitted maps of true magnitude (*M*) and referenced phase angle (*ϕ*) for eikonal waves.

Based on these qualitative results, we fit all data points (of a given arc cluster) to a circle, as represented by the pink curve in Figure 3d. We stress that the center of the arc determines the natural reference for sSNOM calibration, bypassing the necessity to identify and subtract the unknown background signal. The numerical derivative of the phase along the arc with respect to the real‐space coordinate along the eikonal wave propagation directly gives *k*, the propagation constant of the SP wave, with the spatial resolution of the order of twice the maximum pixel size.

To be noted, the total arc spread across the photonic dot is about three‐quarters of π (less than π) in the map shown in Figure 3d. Thus, the overall distance that the SP wave propagates in real space is less than *λ_SP_*/2, which explains why no complete fringe pattern (full period of SP wave) can be observed. For such a sub‐diffractional size of the nano‐disk, *λ_SP_* cannot be determined by any existing technique. Outstandingly, with the new method, not only can we qualitatively reveal the existence of radial surface polaritons by the imaging of the near‐field optical signal in the Argand space, but we can quantitatively measure the SP wavevector and, in principle, map it with the resolution limited by the sSNOM pixel size.

With the new technique, a “tail swirl,” a small segment in the Argand plane due to the propagation of SP in the oxidized edge belt, which falls out of the single arc sequence, can be fitted with a separate SP eikonal wave (light green circle in Figure 3d; Figure S4a). Each eikonal SP has its own reference point (arc center), which means a different optical background for bare silver and for silver oxide materials to be subtracted. There is a large drop in the background across the oxide/bare Ag boundary, even though the sSNOM amplitude is continuous (arc trajectory is not broken). Without new eikonal wave analysis, the sSNOM amplitude or phase, Abs(S_3_) or Arg(S_3_), cannot be used to determine the edge of two distinct materials. The physical origin of the background sSNOM signal is due to the surface impedance of the displacement current flowing from the tip to the sample, neglecting (or averaging out) the SP propagation and interference patterns. Here, a different value of surface impedance of the SiC/2D‐Ag/EG vdWH for bare Ag‐dot and for oxidized silver region is detected, allowing one to clearly trace the oxide layer boundary (Figure 3b,c).

A smaller radius of the oxide SP's arc corresponds to a substantially smaller magnitude of the SP wave, which is the quantitative measure of the lateral field confinement. Upon closer examination, we also noticed that the SP wave exists outside of the photonic dot region with much smaller, though non‐zero magnitude, shown as a short blue arc in the zoomed‐in Argand plot in Figure S4b. This radius of the arc in the SiC substrate, ∼0.002, is far smaller than the SP magnitude in the oxide, 0.396, which is smaller than that in bare 2D‐Ag material, 1.471, at the excitation wavelength of 994 cm^−1^. Thus, the total ratio of the sSNOM field between the photonic dot central region and the substrate outside the oxide belt constitutes >700.

The propagation constant derived with our eikonal phase analysis allows to determine the SP dispersion even in the absence of a clear wave pattern formed, e.g., for confined modes. The flowchart of the analysis is shown schematically in Figure 3, and detailed in Section 4. In brief, each pair of single‐frequency maps (Abs and Arg, panels (b) and (c)) is fitted to a radial eikonal wave and a background component, as shown in panel (d) for a single radial direction (evolution of SP along the radial coordinate from the center of nano‐disk). The fitted parameters for all radial vectors are combined into the new maps for true SP eikonal magnitude and phase (panels (e, f)). Note the sharp boundary in eikonal parameters at ∼250 nm radial coordinate, which corresponds to the inner edge of the oxide belt, as opposed to the broad features in the original data, displaced from the actual belt edge location.

**FIGURE 4:**
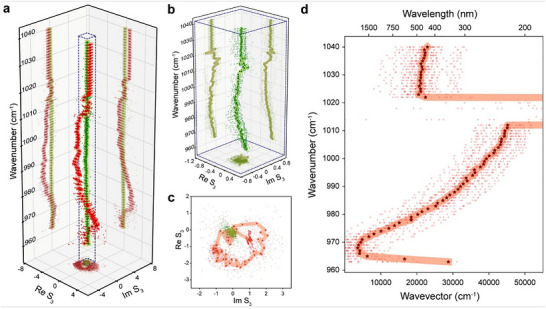
Dispersion of material properties of bare Ag vs. oxidized belt. (a) 3D hyperspectral Argand plot of background (center coordinates) of the fitted bare Ag (red) and oxide (green) cluster arcs from Figure 3d vs. excitation frequency (side walls show projections on Real and Imaginary axes). (b) Zoomed‐in view of the oxide belt data (green) from the box in panel (a). (c) 2D Argand plot of the data in panel (a). (d) Dispersion relation of the SP as computed along different radial directions (the line shows the average dispersion relation for the whole dot).

The background signal (sSNOM reference) varies non‐monotonously with the excitation frequency, as shown in Figure 4a. Figure 4c presents the Argand plot of the frequency evolution of the reference (not to be mixed with the spatial evolution of SP phasor in Argand plots in the rest of the paper). One should expect non‐negligible frequency dependence in the vicinity of the LO mode of the SiC substrate. Although a complicated response of the vdWH with multiple 2D material components needs to be modeled to predict an explicit shape of optical impedance, some qualitative arguments are presented next. We stress that the spectral dependence of the reference is due to the non‐propagating (non‐polariton) near‐field components, the main part of which is the uniform dielectric screening by the sample. If one considers this referencing to be due to the screening of the tip (image dipole) by the vdWH stack, a full 2π cycle of the reference in the complex plane corresponds to passing a pole of the dielectric response function. We observed that the reference (i.e., the central point of the fitted arcs in Figure 3d) for oxide (green cluster) varies much less with the frequency compared to the reference point for bare Ag (red cluster arc), though both have similar spectral features, which becomes clearer in the zoomed‐in image in Figure 4b. While the spectral behavior in the excitation range of 963–1040 cm^−1^ is formed by the optical phonons of SiC, as discussed next, the optical responses of the regions of distinct 2D material – the bare Ag inside the nano‐disk and the oxidized belt – heavily modulate the signal. Being less conductive than metallic silver, the SiC/oxide/EG structure shows less screening, which is consistent with a smaller confined SP amplitude.

The propagation constant for SP was obtained from the eikonal fitting of the hyperspectral cube of sSNOM maps vs. the excitation frequency from 963 to 1040 cm^−1^. The resulting SP dispersion relation for bare Ag is shown in Figure 4d, averaged over different radial directions. The main feature (discontinuity) of the dispersion plot is due to the Reststrahlen band of SiC optical phonon (see also hyperspectral data on “bulk” non‐patterned SiC/2D‐Ag/EG films in Section S9). Notably, similar features appeared in the background signal in panels (a, b) in the same spectral region.

It is instructive to compare the results of the eikonal wave model to the “classical” approach (by counting peaks in sSNOM map to define a wavelength). Although this cannot be done in sub‐diffractional photonic quantum dots of our study. In order to validate the new eikonal technique, in Section S8, we provide a similar study on hBN flakes with the area much exceeding the polariton wavelength. Figure S14 provides the summary of results: the eikonal technique allows to experimentally obtaining the polariton dispersion curve in better agreement with the theoretically predicted one, also using data from a local area, significantly smaller than needed for peak counting (cf. number of pixels used, ∼40 in eikonal model vs. ∼200, as shown in Figure S12b,c).

Further validation of the dispersion of the confined polariton can be obtained from a comparison to the bulk SP modes. In section S9, we present the hyperspectral mapping of non‐structured SiC/2D‐Ag/EG films, that is, the sample which was not used for nanofabrication of plasmonic quantum dots. A common wavelength analysis was not possible for the detection of the SP waves due to the very strong diffraction of the (long wavelength) modes off the surface morphological non‐uniformities. As we discussed in the introduction, standard tools are often not capable of determining the SP wavelength, which motivated us to develop an alternative spectroscopic analytical technique here. The Fourier analysis was implemented to receive some spectral information about bulk SP modes, without spatial resolution (averaged over the area of several sq. micrometers), as detailed in SI. Even though not conclusive, the spectral results on bulk samples can be compared to the dispersion in Figure 4d. In brief, confined and free (bulk) modes share a number of common spectral features (see Figures S17 and S19 and Section S9), due to the same physics of MIR response of SiC/2D‐Ag/EG material. However, it is the eikonal model that allows to get the SP dispersion even for the samples with a complex structure.

**FIGURE 5:**
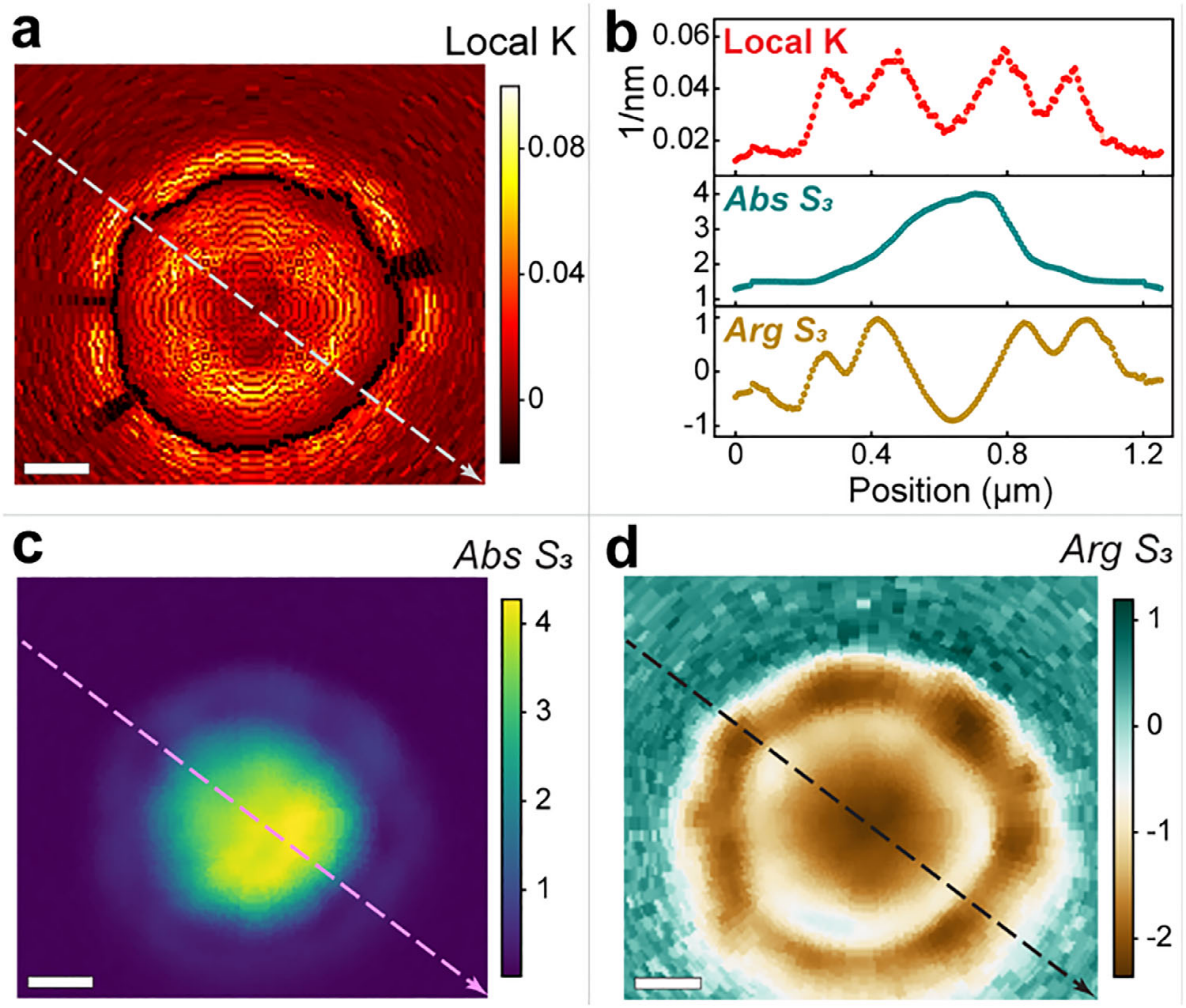
Local propagation constant for confined SP. (a) Calculated map of local propagation constant. (b) Line profiles of the local propagation constant, Abs(S_3_) and Arg(S_3_) (from top to bottom) extracted from the diagonal line in each map. (c,d) sSNOM Abs(S_3_) and Arg(S_3_) maps for comparison. The excitation wavelength equals 994 cm^−1^. Scale bars are 100 nm.

Within the eikonal model, the map of $\frac{\partial \phi }{\partial r}$ shows the local values of the propagation constant of the SP wave (Figure 5a). To verify the applicability of the radial SP wave model, we also derived the other components of the gradient of the eikonal wave (presented in Figure S6a–d in polar, not Cartesian coordinates). Note that all other components of the gradient are much less, at the noise level, compared to the radial propagation constant. The eikonal technique allows for local mapping of the wavevector, independent of the background, as one can see from the line profiles of *k_SP_*, Abs(S_3_), and Arg(S_3_) in Figure 5b, extracted from the line shown in Figure 5a,c,d. The non‐flat shape of the *k*‐profile is expected for a confined mode, being a wave packet, as opposed to the free SP, with a single value of its wavevector for fixed SP frequency.

In this work, a very strong **space localization** ratio was measured for SiC SPs confined by 2D‐Ag/EG: indeed, the photonics quantum dot radius is ∼250 nm < *λ_o_*/40, for *λ_o_* ∼10 µm. The complete SP localization with no traceable field spill‐out outside of the deep‐sub‐wavelength dot shape was achieved: we estimate the true SP wave magnitude ratio >150× to appear between the photonic dot edge (to remember this is natural Ag‐oxide belt area) with respect to the bare SiC substrate outside the confinement area, while an additional factor comes from the ratio between the dot center region and the oxide belt ranging from 3× to 16×, maximized at 973 cm^−1^, around the LO feature of SiC near 966 cm^−1^. This results in an extreme field lateral confinement factor ∼40, along with the large vertical CF = *λ_o_*/*λ_SP_* ∼50. Large CF factors were obtained earlier by detecting the SP wavelength at the edge of other 2D materials: the hBN, Ge_3_Sb_2_Te_6_, and transition metal dichalcogenide samples, as summarized in Table 1.

**TABLE 1:** Confinement factors reported in other 2DMs or 2D vdWHs with MIR excitation.

Material	λ_o_/λ_SP_	Lateral CF	Refs.
Few‐layer MoO_3_	120	—	[21]
hBN/Au grating	132	—	[22]
Monolayer graphene (MLG) in hBN	150	—	[24]
MoS_2_, MoSe_2_, WS_2_, WSe_2_/SiC	up to 190	—	[25]
hBN cones	86	61	[5]
MoO_3_ nano‐ribbon	133	∼60	[15]
MLG/hBN nanodisks	∼100	∼48	[16]
hBN/Au cavity	∼50	∼24	[17]

## 3 Conclusion

In conclusion, an analytical method for sSNOM hyperspectral mapping was developed to reveal the local values of the propagation constant of confined surface phonon polaritons. The method was demonstrated on SP localized in 2D‐Ag/EG plasmonic nano‐disks with sub‐wavelength sizes. Based on the new eikonal wave approximation, we detected an ultra‐strong confinement of SiC polariton waves by 2D‐vdWH‐based photonic quantum dots. The spectral dependence of both the phase velocity (wavevector) and background sSNOM signal (dielectric screening response function) reveals the Reststrahlen band of SiC as the physical origin of the effect. Scanning the trajectory of SP phasors (in Argand diagram of the sSNOM data), we confidently defined the boundary between regions with different material composition of the nano‐disk, the bare 2D‐Ag center, and a belt due to partial oxidation of the silver edge, which cannot be easily revealed by other optical characterization techniques. Finally, the map of local SP propagation constant was obtained, with substantially sub‐wavelength resolution, which allowed us to measure the lateral confinement of vdWH SPs. Extreme values of CF, which are due to just a few layers of a composite 2D material, witness the large photon density of states near the surface, thus opening opportunities for engineering non‐linear and quantum 2D‐photonic devices in the future.

## 4 Methods

### 4.1 Sample Fabrication

The fabrication process is schematically illustrated in Figure 1a. Monolayer epitaxial graphene (EG) was first grown on a 6H‐SiC (0001) substrate (Coherent Corp.) via thermal decomposition. The SiC surface was annealed at 1500°C, 700 Torr in a 10% H_2_/Ar mixture for 30 min, followed by heating to 1800°C in pure Ar (700 Torr, 20 min) to form monolayer EG.

Subsequently, a monolayer of Ag was prepared through confinement heteroepitaxy. The EG surface was treated by O_2_ plasma to generate defects that act as intercalation pathways. The plasma‐treated EG was then placed face‐down in a crucible containing ∼50 mg of Ag powder, followed by annealing at 900°C, 500 Torr, with 50 sccm Ar carrier gas for 1 h, enabling Ag intercalation at the EG/SiC interface.

Circular discs of 2D‐Ag/EG were defined by electron‐beam lithography (EBL). The substrate was spin‐coated with PMMA and baked at 180°C for 90 s, exposed using a Raith EBPG5200 Plus e‐beam system, and developed in a 1:1 mixture of MIBK:IPA for 60 s. The exposed 2D‐Ag/EG regions were etched by N_2_ plasma (20°C, 15 s) in an ULVAC NE‐550 system. Finally, the resist was removed by lift‐off in PRS3000, acetone, and IPA, yielding isolated 2D‐Ag/EG nanodisc arrays.

We assume that the formed 2D Ag is mostly one‐layer based on the predictions carried out in a previous study where the relationship between the number of layers and metal's chemical potential was established based on the first‐principles equilibrium‐phase stability calculations [30]. Advanced calculation predicts formation of two distinct phases with slightly different Ag density, depending on the registration with the underlying SiC lattice [38]. The SiC morphology should also determine the EG layer thickness (up to 2 layers) and continuity. The areas outside the plasmonic nanostructures, after etching, are expected to be bare SiC, as illustrated in the last step of the fabrication process schematic picture.

### 4.2 Sample Characterization

Cross‐sectional specimens were prepared using in situ lift‐out in a focused ion beam (FIB) system (Helios Nanofab DualBeam 660, FEI Inc.). To minimize damage and contamination during milling, an initial ∼20 nm amorphous carbon layer was deposited on the sample surface by sputter coating. Subsequently, an additional ∼400 nm amorphous carbon protection layer was deposited sequentially by electron‐beam deposition, followed by ion‐beam deposition prior to milling. FIB thinning was performed with a Ga^+^ ion beam, starting at 30 kV and gradually reduced to 1 kV to achieve fine thinning of the lamella. High‐resolution scanning transmission electron microscopy (STEM) was performed using an FEI Titan^3^ G2 microscope operated at an accelerating voltage of 200 kV. The probe was configured with a convergence semi‐angle of ∼30 mrad and a probe current of ∼70 pA. High‐angle annular dark‐field images were acquired using a detector with a collection angle range of 51–300 mrad, providing Z‐contrast sensitivity to the atomic structure.

The near‐field imaging was performed using a scattering‐type scanning near‐field optical microscope, a custom‐built Neaspec system. The instrument operated in pseudo‐heterodyne mode with a tapping amplitude of ∼70 nm, using ARROW‐NCPt probes from Nanoworld (tip radius < 25 nm). For hyperspectral mapping, continuous‐wave quantum cascade laser excitation (MIRCat, Daylight Solutions) was used in the range of 963–1040 cm^−1^ (10.384–9.615 µm), with power kept below 2 mW at the focal aperture.

### 4.3 Phase Analysis of SP Dispersion

A hyperspectral cube of sSNOM maps vs. the excitation frequency from 963 to 1040 cm^−1^ (see Table 1) was carefully registered for batch processing. For each excitation wavelength, we obtain the reference point/background signal and the propagation constant for each of 3 separate regions: bare 2D‐Ag central area, oxide layer belt, and SiC substrate (highlighted in red, green, and light blue in Figure S4). For the axial symmetry of the photonic dot, which yields the radial eikonal wave, we transform the real‐space image from the Cartesian into polar coordinates. We generate the phasor map in Argand space. Within the nano‐disk, data points with the same *r* but different *θ* (polar coordinates) have approximately the same phase (belong to the single circular wavefront), compared to Figure 2f. The data points with the same *θ* must show fast phasor rotation with *r*, as highlighted in the Argand plot (Figure 2e) by purple scatter points, corresponding to the green rectangular region in the real space map, see Figure S2a. Radial direction of the maximum variation of the phase is used to compute the SP propagation constant, noting a slow variation of the amplitude due to edge‐induced perturbation.

The fit for the radial direction slices (both red and green data points in Figure 3d) is performed to confine data to a circle in the Argand space. The turning point between two arcs, or preferably, the point where the change of phase flips the sign (with respect to the center of the red arc), defines the exact position of the boundary between pure 2D‐Ag and oxidized edge belt (shown as magenta curve in Figure 3b). The center of the fitted circle is the referencing point for each region. Note that the references of different regions vary for different excitation wavelengths.

Having a reference point allows to determine the true phase (and magnitude) of the phasor of the surface wave and overcome the ambiguity of proper referencing/normalization of sSNOM signals. This yields mapping of *M* (Figure 3e), and *ϕ* (Figure 3f), the eikonal wave magnitude and phase, to the real‐space polar coordinates *θ* and *r*, and stacks it vs. the excitation wavelength for studying the SP dispersion. The gradients of the phase matrix with respect to the *θ* and *r* will provide the propagation constant in the chosen direction at each location in real space, at the fixed excitation wavelength. For the radial eikonal wave, the radial component of the gradient gives the wave vector of the polariton, while the other component of the gradient is negligible.

Similar eikonal analysis applied to sSNOM data collected from a series of photonic quantum dots of variable radius showed a close correlation for the measured propagation constants, as shown in Figure S8.

## Funding

This work was funded by NSF award DMR2011839. NSF cooperative agreement DMR‐1539916 and DMR‐2039351. Air Force Office of Scientific Research (AFOSR) FA9550‐19‐1‐0295. NSF award ECCS‐2025462.

## Conflicts of Interest

The authors declare no conflicts of interest.

## Supporting information




**Supporting File 1**: adma73494‐sup‐0001‐SuppMat.docx.

**Supporting File 2**: adma73494‐sup‐0002‐VideoS1.mp4.

## Data Availability

The data that support the findings of this study are available from the corresponding author upon reasonable request.;
