# Supplementary material for: Ultra‐Confinement of Polaritons in Single Atomic Layer Ag Photonic Quantum Dots
Source: Adv Mater. 2026 May 26;38(36):e21015. doi: 10.1002/adma.202521015 (PMC13310107; doi:10.1002/adma.202521015)
Supplement: Supplementary file 1 — Supporting File 1: adma73494‐sup‐0001‐SuppMat.docx. [file ADMA-38-e21015-s001.docx]

Supporting Information

Ultra-Confinement of Polaritons in Single Atomic Layer Ag Photonic Quantum Dots

Xinyi Li, Tetyana Ignatova, Chengye Dong, Krishnan Mekkanamkulam Ananthanarayanan, Rinu Abraham Maniyara, Arpit Jain, Furkan Turker, Vinay Kammarchedu, Aida Ebrahimi, Joshua A. Robinson, and Slava V. Rotkin*

X. Li, J. A. Robinson, S. V. Rotkin

Department of Engineering Science and Mechanics, The Pennsylvania State University, University Park, PA, USA
E-mail: rotkin@psu.edu

T. Ignatova

Department of Nanoscience, Joint School of Nanoscience and Nanoengineering, the University of North Carolina at Greensboro, Greensboro, NC, USA

C. Dong, J. A. Robinson

Two-Dimensional Crystal Consortium, The Pennsylvania State University, University Park, PA, USA

C. Dong, K. M. Ananthanarayanan, R. A. Maniyara, A. Jain, F. Turker, A. Ebrahimi

Department of Materials Science and Engineering, The Pennsylvania State University, University Park, PA, USA

V. Kammarchedu, A. Ebrahimi

Department of Electrical Engineering, The Pennsylvania State University, University Park, PA, USA

A. Ebrahimi

Department of Biomedical Engineering, The Pennsylvania State University, University Park, PA, USA

J. A. Robinson

Department of Chemistry, The Pennsylvania State University, University Park, PA, USA

J. A. Robinson, S. V. Rotkin

Department of Physics, The Pennsylvania State University, University Park, PA, USA

**Table of contents:**

S1. Near-field imaging of fabricated 2D-Ag/EG plasmonic nano-disks

S2. Correlated arc shape in Argand space: indication and verification of surface polariton wave

S3. Referencing point: background screening by different material compositions

S4. Dispersion relation of polaritons

S5. Characteristics of eikonal waves

S6. Evaluation of eikonal model

S7. Validation of eikonal model results

S8. Validation of eikonal model analysis with hBN/Gr/hBN polaritons

S9. Polariton dispersion in non-structured SiC/2D-Ag/EG films

**S1. Near-field imaging of fabricated 2D-Ag/EG plasmonic nano-disks**

The studied 2D-Ag/EG plasmonic nanostructures on the SiC substrate are fabricated by the following process. The EG is firstly grown on SiC substrate by silicon sublimation on the Si face of 6H-SiC (0001). Then, the intercalation process happens through thermal evaporation: vaporized Ag atoms diffuse in between the EG layer and SiC substrate and form a crystalline 2D-Ag layer. Afterwards, a mask with designed plasmonic shape will be formed by e-beam lithography and the areas outside the masked region will be removed by the chemical etching process. Finally, we will obtain 2D-Ag/EG plasmonic nanostructures (photonic dots) on the SiC substrate.

The EG sample, formed upon epitaxial growth process, may include a partial monolayer EG on top of the buffer graphene layer. The 2D-Ag layer will form between the buffer graphene layer and SiC substrate during the intercalation process. Therefore, the Ag layer is encapsulated by the monolayer graphene and partial two-layer graphene in different areas. The substrate outside the plasmonic nanostructures is expected to be bare SiC.

The area we studied consists of a series of disk-shape 2D-Ag/EG nanostructures, where we performed near-field imaging. Representative maps at the excitation wavelength 968 cm^‑1^ are shown: sSNOM/optical maps, Figure S1c-j, were taken simultaneously with the mechanical/AFM channels, Figure S1a-b. From AFM topography and (mechanical) phase image, we can distinguish the terrace step edges of SiC substrate as well as the shape of the nano-disks. The size of the nano-disks is below 1 μm, increasing from left to right.

The “snake-like” morphologies within the nano-disks have 0.7 nm height variation that are consistent with the material composition of incomplete two-layer EG. In the course of metal interaction, monolayer or bilayer of quasi-free-standing graphene are formed from a buffer or a buffer plus a monolayer EG. In case the initial EG contains an incomplete monolayer, the resulting graphene composition after Ag intercalation will be less than 2 and greater than 1 layer, thus forming irregular partial bilayer structure.

Figure S1c-j shows the simultaneously obtained multichannel sSNOM optical maps, i.e., different harmonic demodulated (1^st^ to 4^th^) sSNOM signals: the optical amplitude (Abs(S_1_) to Abs(S_4_)), and the optical phase (Arg(S_1_) to Arg(S_1_)) of a series of nano-disks. Upon visual inspection, we notice that the region inside the nano-disk shows a higher optical amplitude signal compared to the outside region (substrate). Also, comparing the phase and amplitude maps, we trace a wide-belt region around the circumference of the nano-disk: it has lower signal, though much higher than of the substrate in all optical amplitude maps, and shows a clear contrast compared to both the region inside the nano-disk and the substrate in all optical phase maps. Need to mention that the optical response we observed in these fabricated nano-disks does not follow nor has a substantial dependence on the surface morphology of the sample, i.e., the near-field signal is not substantially affected by the existence of impurities/residues, induced during the fabrication/etching process. Obviously, there is no clear trace of wave patterns formed within the region of nano-disk nor in the outside region of the nano-disk, which is easily explained by having about four order of magnitude difference in scale between the disk size and laser wavelength.


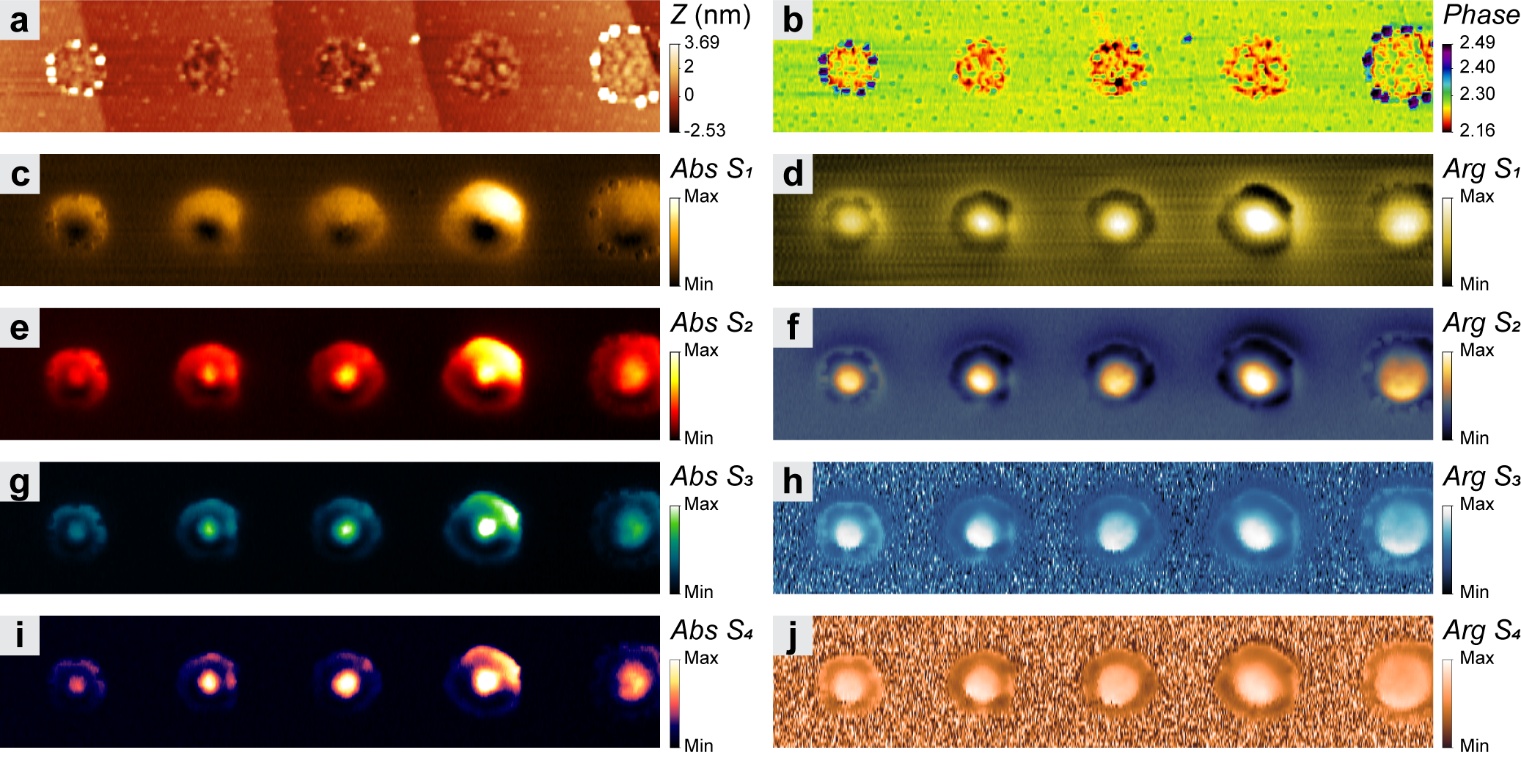


**Figure S1.** Multichannel sSNOM maps of 2D-Ag/EG plasmonic nano-disks. Scale bar 500 nm.

Hyperspectral sSNOM mapping was performed with excitation wavelength 963-1040 cm^‑1^ of the region of interest, i.e., the third disk counted from left in Figure S1a. The representative sSNOM Abs (S_3_) and Arg (S_3_) maps are shown in Table S1 and MP4 S1.

**Table S1.** Hyperspectral sSNOM maps of the region of interest.


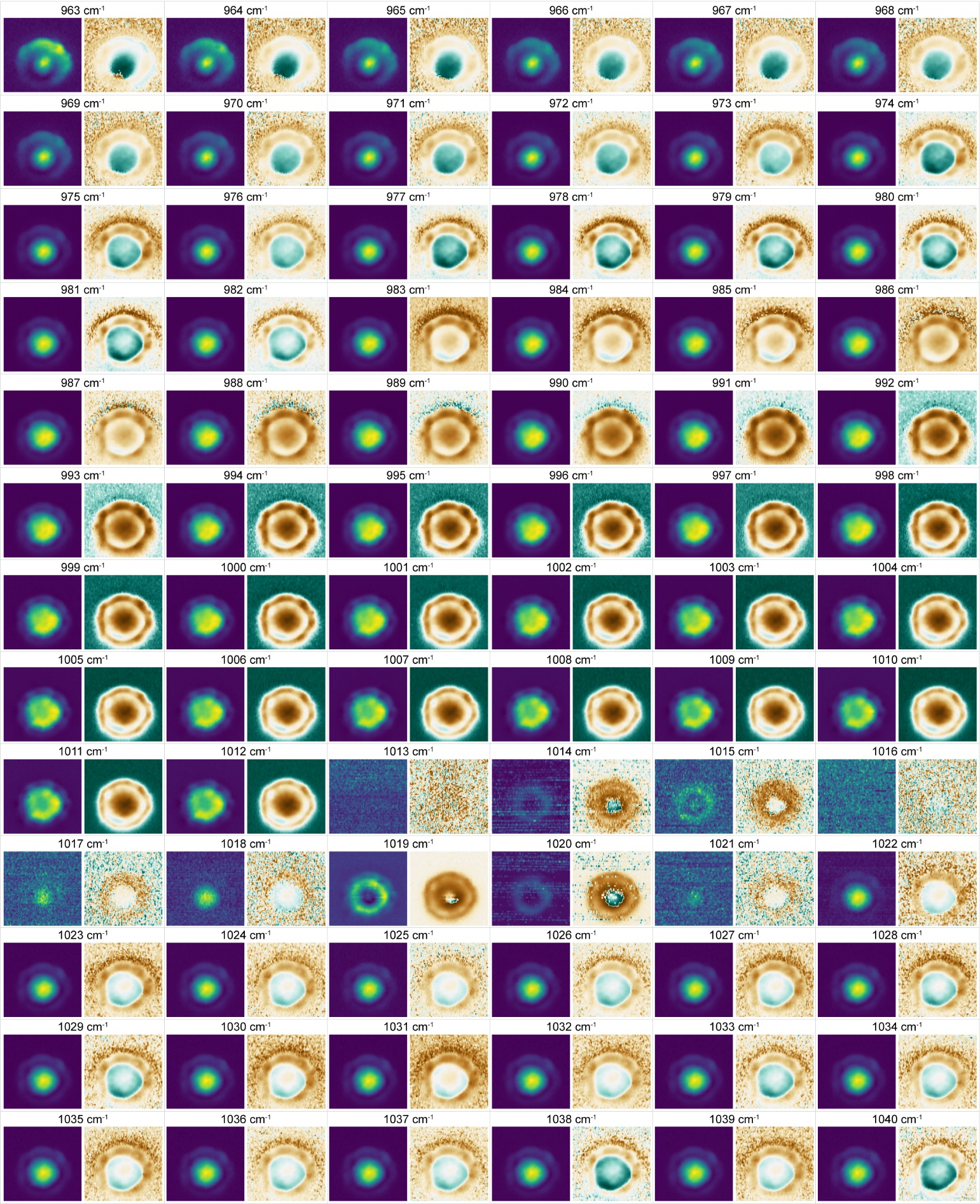


**S2. Correlated arc shape in Argand space: indication and verification of surface polariton wave**

The near-field optical signals we obtained from sSNOM measurements are complex valued: $S_{3}=Ae^{i\varphi}$, where the optical amplitude is $A=Abs\left( S_{3} \right)$ and the phase $\varphi=Arg\left( S_{3} \right)$. One can represent the sSNOM complex valued response by the real ($Re S_{3}=Acos\varphi$) and imaginary part ($Im S_{3}=Asin\varphi$).

Figure S2a is the cross-correlation plot between the imaginary and real parts, where the gray scatter dots are the S_3_ data, taken at excitation wavelength 1030 cm^‑1^, from the whole region of interest. This panel reproduces the Figure 2e in the main text. Figure S2b-c are obtained by the Cartesian-to-polar coordinate transformation of the third demodulated harmonic optical amplitude, Abs(S_3_), and phase, Abs(S_3_), maps of the region of interest from raw sSNOM data. The blue scatter dots in panel (a) represent the data from a single slice (with 4 pixels in $\theta$ direction) of the polar maps, highlighted by green rectangles in Figure S2b, c. Two adjoint arcs can be clearly traced (blue scatter dots). The shape of an arc in the complex plane corresponds to an eikonal wave with the constant amplitude and variable phase of a phasor of the sSNOM signal, indicating the existence of a propagating surface polariton (SP) wave.


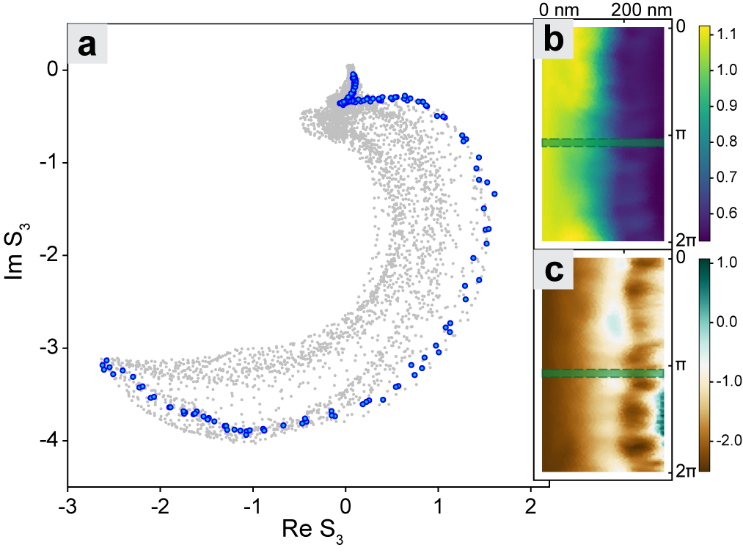


**Figure S2.** Correlated arc shape in Argand space.

To further verify the existence of radially propagating surface polariton wave, we focus on a single arc in Argand space and trace the rate of phase variation along the arc with respect to spatial displacement of their corresponding locations in the real space map. To determine its propagation direction, in Figure S3a we choose in Argand space a sequence of segments (marked by the rainbow colors) with the fixed amplitude (same as the radial coordinate for the spokes from the center of the arc). The Figure S3b shows that the trajectory, from red to magenta, moves from the disk center to its edge in the real space. For an outgoing radial wave we expect the counterclockwise evolution of the phasor in Argand space (the clockwise evolution would correspond to inward radial wave). Indeed, taking a sequence of trajectory points/small regions in the real space displaced in radial direction, as shown in Figure S3d, their counterparts in Argand space follow the counterclockwise arc, plotted in Figure S3c, which supports our assumption.


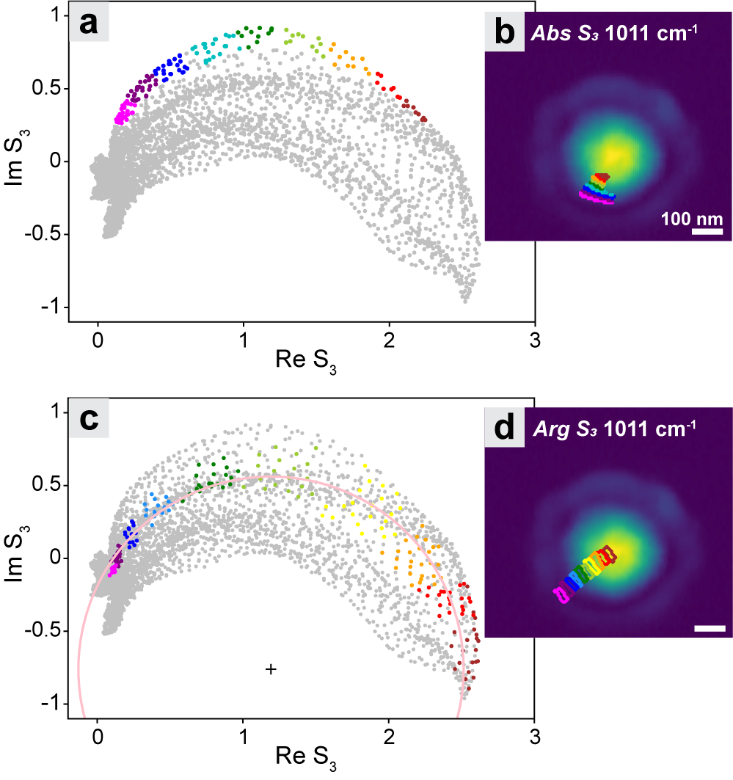


**Figure S3.** Correlating trace of phase in Argand space vs. polariton wave in real space. (a) Selected clusters in Argand space with variation in phase but nearly equal amplitude. (b) The corresponding data points in real space form a radial wedge of the outgoing radial wave. (c) Data points in Argand space from the sectors selected in (d). All the selected data points are fitted to a circle (pink curve) with the center marked as a black cross. (d) Areas selected in the real space along a single radial direction.

**S3. Referencing point: background screening by different material compositions**

The sSNOM signal we obtained consists of (1) a propagating wave component, stemming from the near-field response of propagating surface polaritons, possibly confined in the photonic dot, and (2) the background signal or non-propagating component, of which the majority is due to the dielectric screening by the spatially uniform sample material. Therefore, to quantitatively measure the eikonal wave properties, such as the wave amplitude and wavevector of the surface polaritons, proper referencing needs to be performed first.

In our new method, the referencing is done by fitting the eikonal arc segment with a circle in the Argand space, where the coordinates of the center of the circle correspond to the complex valued background screening by the material, while the radius of the circle is the true magnitude of the eikonal wave and the phase velocity (the rate of change of the true phase) reveals the propagation constant of the wave.

Besides the larger arc (represented by red scatter dots), we notice a “tail swirl” (represented by green scatter dots), a small segment in the Argand plane which clearly falls out of the single arc sequence in Figure S4a. Clearly, this segment possesses a different referencing point compared to the red arc, as well as a different magnitude (radius of the circle) of the wave. We fitted this segment of the arc (green scatter dots) in Figure S4a. The smaller radius of arc corresponds to substantially smaller magnitude of the radial wave in this spatial location. Specifically, the green dots data come from the belt region near the circumference of the nano-disk. We assume it is composed of oxidized 2D-Ag. We note that the eikonal waves confined within the belt and the nano-disk propagate in the same (outward radial) direction (which can be seen from the counterclockwise evolution of the phasor). The smaller magnitude indicates the weak “leak” of the eikonal wave from bare 2D-Ag center to oxide belt, which results in substantial drop of the wave magnitude (and therefore, the EM field energy).


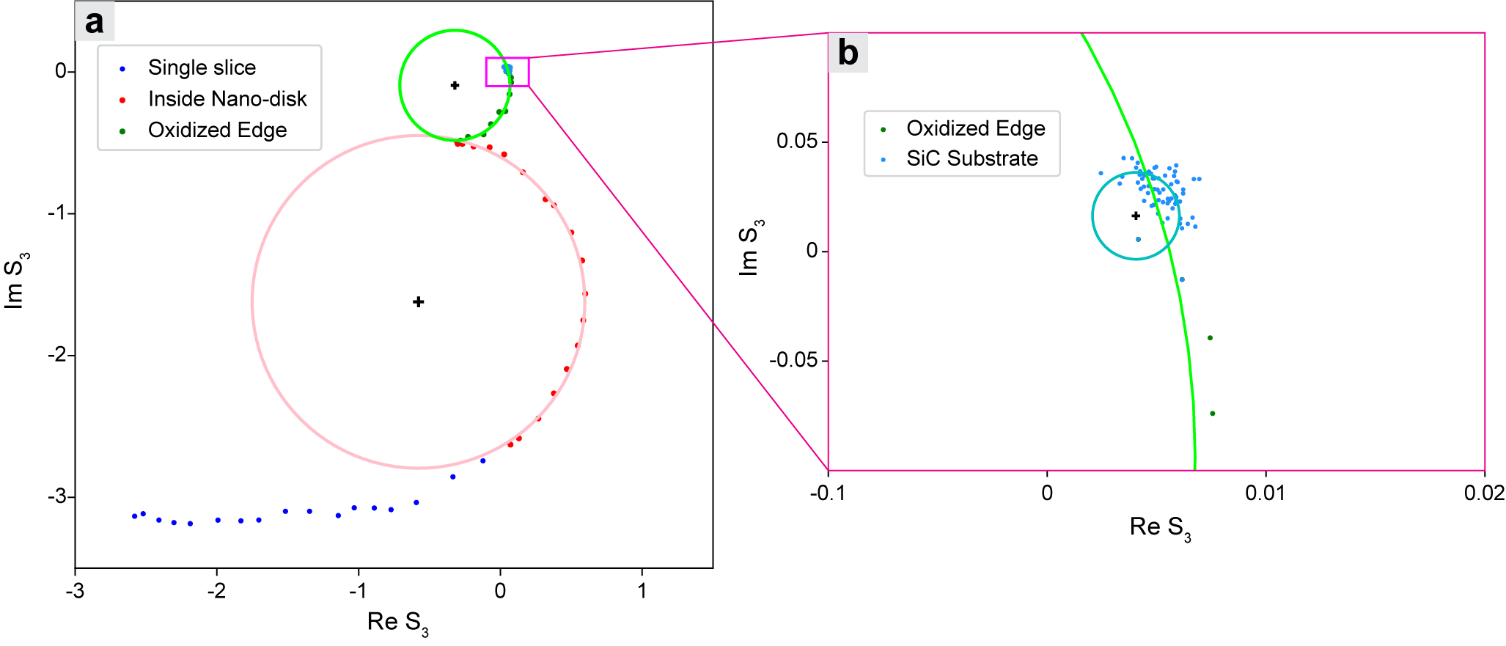


**Figure S4.** Fitting eikonal wave arcs for 3 different regions. (a) Main part of the Argand plot shows the arc due to the bare Ag material in the central region of the photonics quantum dot (pink) and the oxide belt (green) – both encapsulated by the EG layer; (b) a magnified view of the last segment of the phasor curve shows the arc for the bare SiC substrate (cyan) (part of the oxide belt data is also shown).

Besides these two arcs, corresponding to bare Ag and oxide belt, we notice another short arc segment at the end of “tail swirl”, which can be seen better in the zoomed-in Argand plot (Figure S4b). We can attribute these data points to the region outside of nano-disk, that is to the eikonal wave on the SiC substrate. The radius of bare SiC arc (cyan) in Figure S4b is far smaller than the radius of light green circle, thus, confirming much smaller true magnitude of the wave outside of the quantum dot and proving our statement on the complete confinement od SP within the nano-disks.

We emphasize that these different arc segments also correspond to different reference signals, which means that the different optical background needs to be subtracted to obtain the true phase of eikonal wave. Since the referencing point (background) is due to the dielectric screening of the tip dipole (in the tip-sample image dipole model), it indicates the different material composition in these regions.

The coordinates of these referencing points (the backgrounds) vary with respect to the excitation frequency. This is due to the material optical response depends on the excitation (Figure 4a). Both the region inside the nano-disk (SiC/bare 2D-Ag/EG) and in the belt (SiC/oxidized 2D-Ag/EG) show dispersion, typical for a response near the frequency pole. By comparing the pole position to the SiC response function we deduce that the non-monotonic behavior is due to the optical phonon of the latter.

**S4. Dispersion relation of polaritons**

In order to determine dispersion of the eikonal wave, the real-space image for each excitation wavelength has been transformed from cartesian into polar coordinates. Then, the angular slices with fixed width (data for a narrow region of angles near the $\theta$ value) are fitted to the arc segments: the center (reference point) and the radius (true wave magnitude) in Argand space are defined (Figure 3). The evolution of the true phase is determined vs. the radial coordinate, $r$, which is limited to the geometrical region of the nano-disk, approximately $r$<260 nm. The overall arc length in the Argand space, $\phi_{tot}=\phi\left( r_{max} \right)-\phi\left( r_{o} \right)$, for each slice is computed and related to the corresponding trajectory length in the real space, $L=r_{max}-r_{o}$, which allows to define the mean eikonal wavelength of the surface polariton within this arc segment:

$$\Lambda=\frac{2\pi L}{\phi_{tot}}$$

The SP eikonal wavelength is defined for each excitation frequency to produce the dispersion relation.

Consequently, we can plot excitation frequency vs. average polariton propagation constant, obtained as: $k_{tot}=\frac{\phi_{tot}}{L}$. Figure 4d shows the dispersion relation obtained within the nano-disk (i.e., using the red scatter dots from the major arc segment in Argand space) and averaged over the angular coordinates. The red markers represent the mean values of wavevectors for each excitation wavelength. We clearly see two branches of surface phonon polariton originated from SiC, denoting the Reststrahlen band of SiC optical phonon. This dispersion relation obtained from the overall arc length $\phi_{tot}$ is an integral property of the disk with the size $L$. Notably, the arc length is not just a linear function of $L$ (see Supplementary Section S5).

**S5. Characteristics of eikonal waves**

Since we have already established radial propagation direction of the polariton wave, we can proceed with selecting the appropriate segments in the real-space map, starting from the center towards the edge of the nano-disk, and find the corresponding clusters in Argand space.

Notice that in Figure S3c, the clusters in the Argand space are spread less tight for the location near the edge of the nano-disk, while they distribute more widely for the clusters near the center, e.g., data points from the magenta cluster show less fluctuation of the eikonal wave magnitude, $M$, than those from the dark red cluster. Thus, eikonal wave is better defined for the magenta segment, compared to the red segment. Partially, this may be due to the transformation to radial coordinates must reduce the angle/radial pixel accuracy near the coordinate origin. Additionally, the arc length of the phase varies from segment to segment in the Argand space, despite the same size of each segment in the real space, indicating the nonlinear change of the true phase with the propagation distance, i.e., the locally varied propagation constant of the eikonal wave.


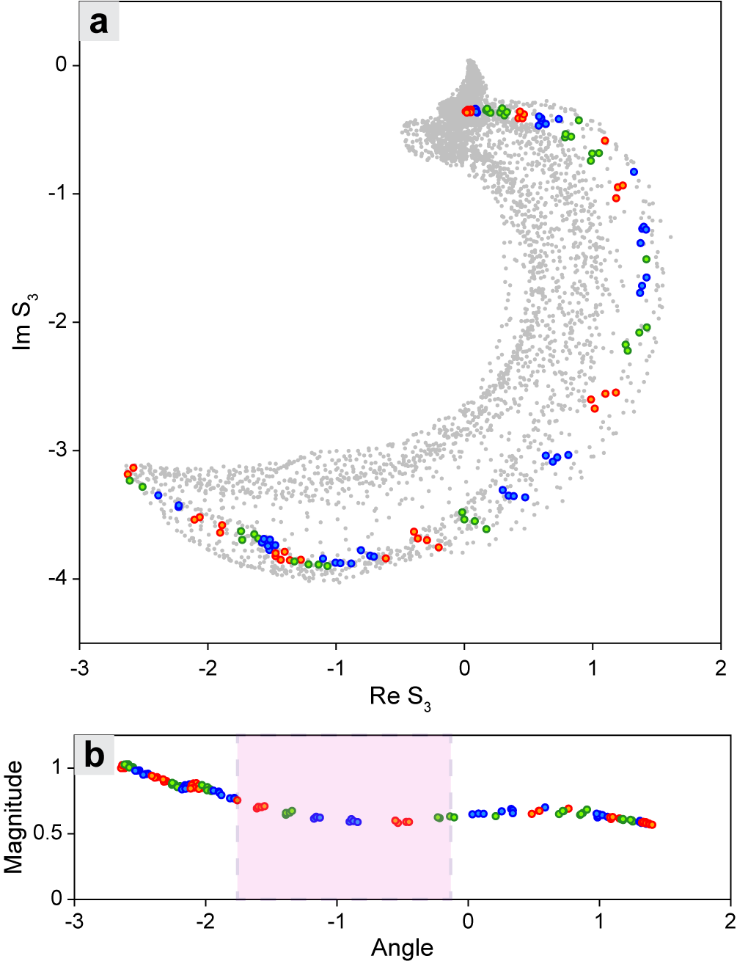


**Figure S5.** Arc fitting: the phase velocity as a function of the phase, proportional to the propagation length, $L$.

To further examine the behavior of the surface polariton waves, we can transform the Argand plot (in polar coordinates) into the true magnitude and true phase coordinates after fitting the scatter dots to a circle and subtracting the reference center, for both red and green arcs. Figure S5b shows the true magnitude and angle from the data points along a specific radial direction in real space, where we can clearly observe the damping of the eikonal wave amplitude towards the edge. However, since the damping parameter is small we will neglect this for the short distance from the disk center to the edge when fitting the radial waves for the rest of the work.

Figure S5a shows the representative plot in Argand space for a specific angular slice in real space at excitation wavelength 994 cm^‑1^. The data points (marked with alternating color) are taken from the small radial boxes of the same size, i.e., for the constant increment of radial displacement in the real space. Using this construction, one can compare corresponding arc length in the Argand space as a function of radial distance from the disk center. The length of the arc segments near the middle of the range (highlighted by pink rectangle in Figure S5b) is larger compared to the ends of the arc, which can be better observed in the plot of the true magnitude and angle (Figure S5b). This hints that the propagation constant of the surface phonon polariton may be a function of coordinates. The true phase gradient (derivative along the correlated arc in the Argand space with respect the trajectory displacement in the real space) is computed for each point/small domain on the sSNOM map, as shown in next section.

**S6. Evaluation of eikonal model**

Figure S6 shows the representative gradient maps of true magnitude and true phase with respect to the $\theta$and $r$ at the excitation wavelength 994 cm^‑1^. As we can see, the values of the angular gradients: $\frac{\partial M}{\partial\theta}$ (Figure S6a) and $\frac{\partial\phi}{\partial\theta}$ (Figure S6c) are close to zero, indicating a little evolution in angular direction, which is consistent with the wavefront of the eikonal wave in the real space being nearly circular. The map of $\frac{\partial M}{\partial r}$ (Figure S6b) evaluates the deviation of the sSNOM data from our assumption of an ideal eikonal wave, i.e., it shows how close is the arc shape to the circle. Including distance dependence of the wave magnitude, the phasor can be represented as following:

$$A_{o}e^{iks+log\frac{M\left( s \right)}{M_{o}}}$$

where $M_{o}$ is the extracted magnitude in Argand space of a data point taken as the wave origin (the center of nano-disk in real space in this case). Naturally, the second term in the exponent gives a logarithmic derivative component to the calculated propagation constant. Figure S5b shows that the true magnitude may deflect from a constant value for some slices (this effect is beyond the oxide belt or SiC region, discussed earlier). However, the variation of the gradient $\frac{\partial A}{\partial r}$ (observed in the map in Figure S6b) is small compared to true phase term.

Within these approximations, the map of $\frac{\partial\phi}{\partial r}$ (Figure S6d) shows the local values of the propagation constant of the eikonal wave.


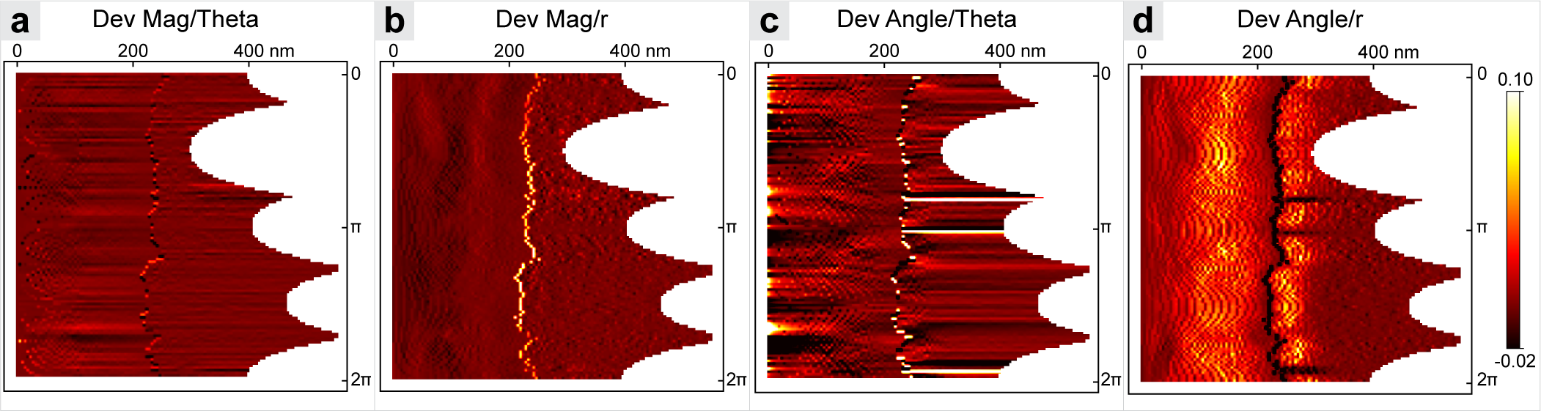


**Figure S6.** Evaluation of an accuracy of the radial eikonal wave model: the maps of all components of the phase velocity gradient. The map of derivative of magnitudes with respect to (a) angular and (b) radial direction. The map of derivative of angular coordinates with respect to (c) angular and (d) radial direction.

**S7. Validation of eikonal model results**

Figure S7 shows the AFM and sSNOM maps of 5 more photonic Quantum Dots (pQDs), along with the one used for main text analysis (disk 13 in yellow rectange). All disks have different diameters, though made of the same material. Obviously, all pQDs demonstrate a similar ultr-strong polariton confinement, which is further supported by taking the data for the eikonal analysis.


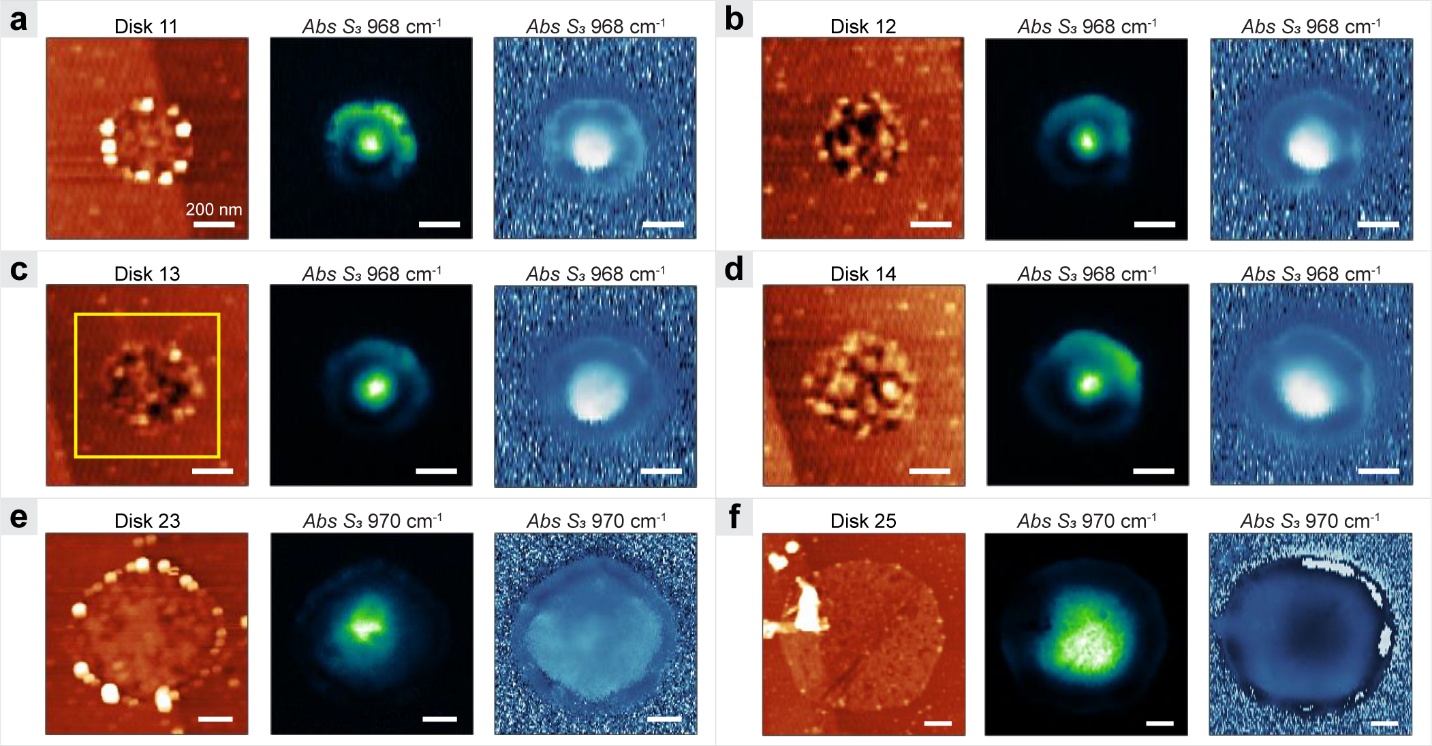


**Figure S7.** sSNOM maps of 2D-Ag/EG plasmonic nano-disks with different sizes. AFM topography image, sSNOM Abs(S_3_), and Arg(S_3_) maps at excitation wavelength 968 cm^-1^ of nano-disk 11-14 (a-d); AFM topography image, sSNOM Abs(S_3_), and Arg(S_3_) maps at excitation wavelength 970 cm^-1^ of nano-disk numbered 23, 25 (e, f). Scale bars are 200 nm. The nano-disk studied in the main text is marked with yellow square in panel (c).

The sSNOM maps from Figure S7 were processed by the same eikonal wave analysis as in main text and resulting propagation constants are plotted vs. the measured pQD diameter (inverse of) in Figure S8. Overall, a clear correlation of the propagation constants for pQDs of a similar diameter is seen, with very little variability, along with some monotonic dependence on the pQD size. Notably, the periodicity (wavelength) is smaller than the disk diameter for large enough disks.


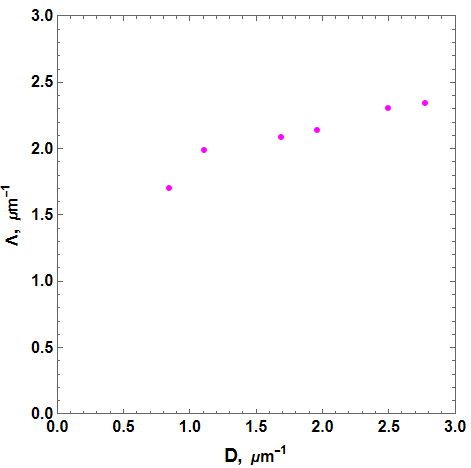
**Figure S8.** Propagation constants for series of pQDs of different diameters. Measured inverse wavelength (propagation constant) for pQDs #11-14 and #23, #25 from Figure S7 obtained from the raw sSNOM Abs(S_3_) and Arg(S_3_) maps by the eikonal wave analysis.

The sSNOM mapping data might potentially contain measurements artifacts, mostly due to a mechanical AFM tip motion instability when moving over a large surface impurity. Since the surface morphology of the pQDs is not perfect (due to the fabrication process), we took additional care to exclude cases that might produce ambiguous eikonal wave analysis. Figure S9 provides examples of “negligible” and “non-negligible” effect of surface impurities on the optical sSNOM signal. Panel (a) shows AFM maps of 5 pQDs, including the one which is used in main text analysis. Panels (b-e) show 4 representative profiles taken along the arrows labeled with the corresponding numbers in the panel (a). In all 4 examples, neither Abs(S_3_) nor Arg(S_3_) signals (green and blue lines) show substantial correlation with the AFM profile (brown line). On contrary, last example in panel (f) shows significant correlation between AFM and Arg(S_3_) channels and should be excluded from the analysis. Note that the height of the impurity in this case exceeds 6 nm, in contrast to 1-2 nm in other cases.


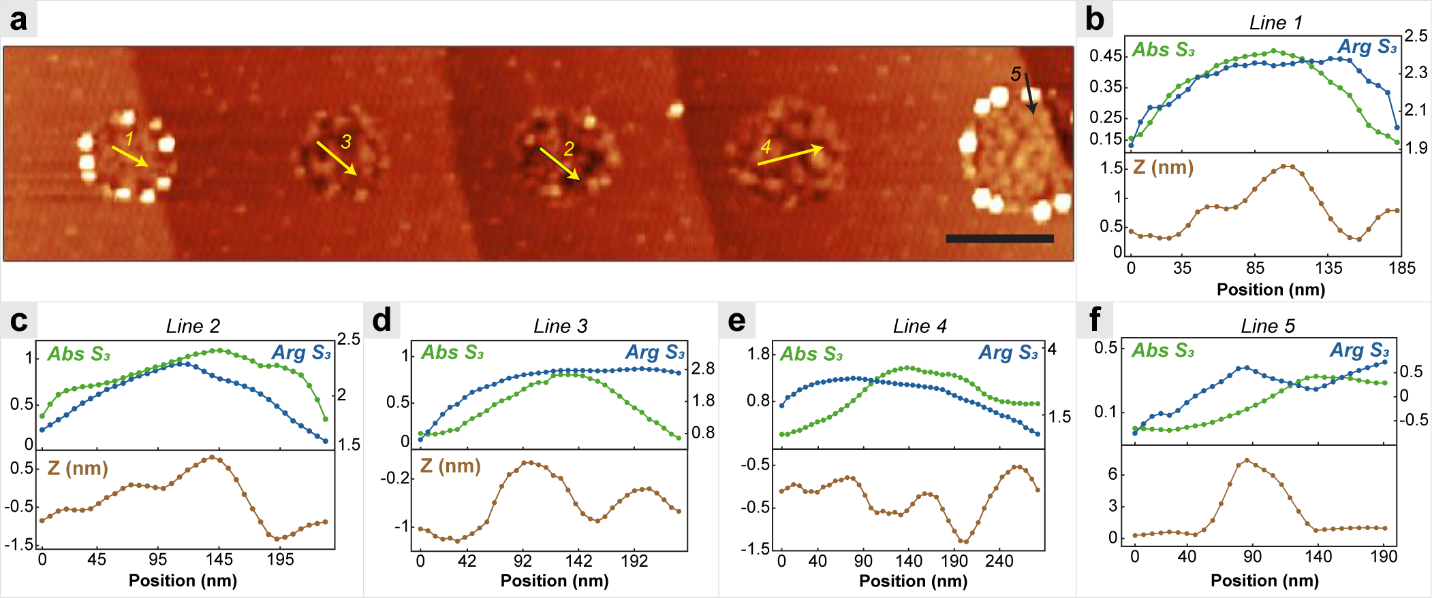
**Figure S9.** Line profiles of sSNOM signals compared to the AFM topography. (a) AFM topography image of nano-disk 11-15. The locations of the corresponding line profiles shown in panel (a-f) are indicated by arrows. The scale bar is 500 nm. (b-e) Line profiles of the sSNOM Abs(S_3_) and Arg(S_3_) (top graph), compared to the AFM Z height (bottom graph) of lines 1-4, where the sSNOM signals show no correlation with the AFM Z height. (f) Line profiles of the sSNOM Abs(S_3_) and Arg(S_3_) (top graph), compared to the AFM Z height (bottom graph) of line 5, where the sSNOM Arg(S_3_) shows some correlation with the AFM morphology due to an impurity with a large height profile.

**S8. Validation of eikonal model analysis with hBN/Gr/hBN polaritons**

To provide additional evidence for legitimacy of novel eikonal analysis we present here a hyperspectral mapping series on another polaritonic material (unrelated to main study on SiC polariton confinement). In this SI section we provide analysis of polariton modes‘ dispersion in hBN/Graphene/hBN sandwich, transferred onto Si/SiO_2_ substrate. The sample was prepared with standard transfer methods: the bottom inset in Figure S10a shows schematically the Si-substrate with 285 nm oxide layer, (thick) bottom layer of hBN, followed by monolayer graphene (MLG) and another (thin) top layer of hBN, corresponding to the cross-section along dashed line in (a). The structure was thouroughly characterized by Raman microscopy – Figure S10e shows Raman signature bands for each layer of the sandwich material. We focus next on the edge area, where the (conformal) film of top-hBN/MLG falls of the cliff of (thick) bottom layer of hBN to the bare SiO_2_/Si substrate (as moving from left to right along the dashed red line in Figure S10a). In this area we expect strong reflection of polaritonic waves from the edge.


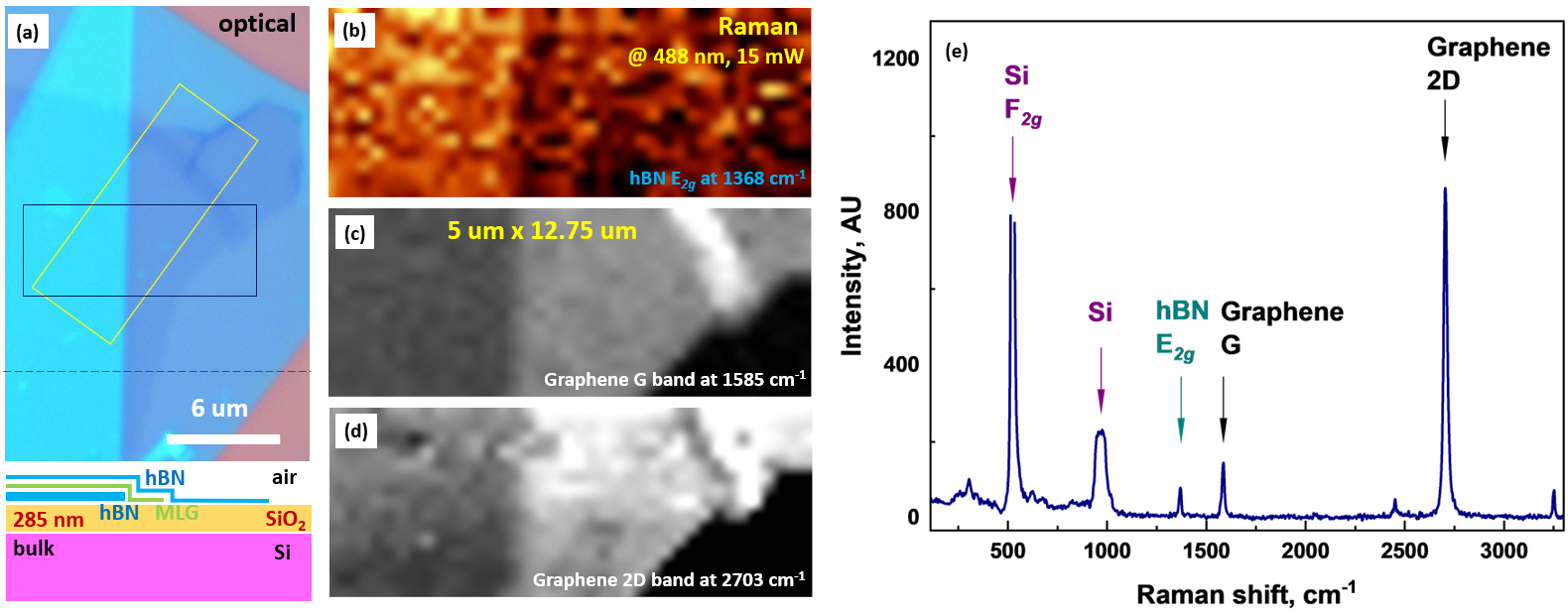


**Figure S10.** Raman characterization of the sample SH4. (a) Optical image of the sample; (bottom inset) the cartoon schematics of the transferred layers as appear along the chosen cross-section (red dashed line) in the micrograph. (b-d) Representative Raman maps to identify graphene (Gr) and hBN layers. The maps are taken over the same area 12.75um x 5um, shown as a black rectangle in (a). The maps are shown for position of characteristic peaks of (b) E_2g_ band of hBN, (c) G and (d) 2D bands of GR, as selected from (e) the Raman spectrum. These peaks were clearly identified in the range 250-3600 cm^-1^. Excitation wavelength was 488 nm.

Figure S11 shows the sSNOM maps of top-hBN/MLG/b-hBN/SiO_2_/Si sandwich material, where the hyperspectral series SH4 (11 single laser frequency maps) were taken. Abs and Arg of S_2_ sSNOM signal at 1410 cm^-1^ are presented in Figure S11b,c. Physical structure is well resolved, including the edge between the left region of full sandwich structure (t‑hBN/MLG/b‑hBN/SiO_2_/Si) and the right region with missing bottom hBN layer (t‑hBN/MLG/SiO_2_/Si). Graphene folds are also clearly resolved in the dark area in Figure S11b.

More detailed mapping was done over the area next to this edge, as shown in Figure S11d. Here Abs S_3_ sSNOM signal is used, the same which is analyzed in the eikonal wave model next. An “ordinary” method of obtaining the polariton dispersion is to count the distance between the maxima of the wave pattern – those are indicated by arrows in panel (d). Notably, the distance between the peaks is not necessarily uniform which makes a large uncertainty for polariton dispersion (to be discussed below). For the sake of convenience of further data analysis, we performed a numerical transformation for hyperspectral map series to make the edge line approximately vertical (since we use raw data, without any interpolation, an exact alignment is limited by the width of the pixel in horizontal direction, which was chosen to be 8 nm in our case). The pre-aligned data is shown in panel (e); the map corresponds to the same height area as in panel (d) with the rugged edge (not shown), as needed for the alignment.

**
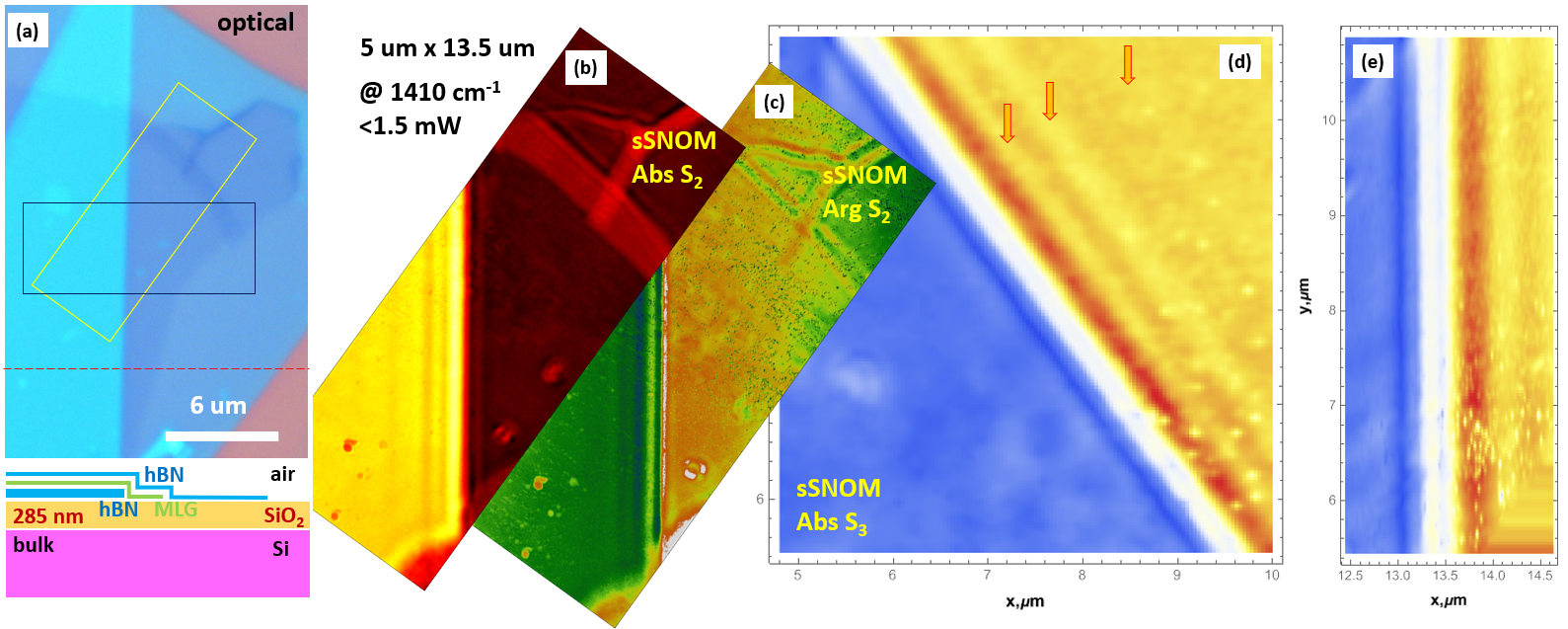
**

**Figure S11.** sSNOM mapping of the hBN polaritons. (a) same optical micrograph as in Figure S10. (b-c) Maps of the area shown by yellow rectangle in (a): O2A and O2P channels are presented. sSNOM contrast clearly distinguishes the edge of thick bottom hBN layer in a good correlation with the Raman maps (cf. Fig. S10b); in (b) sSNOM also reflects graphene folds in the top of the map (cf. Fig. S10c). (d-e) Maps of O3A at 1410 cm^-1^. Arrows in (d) indicate maxima of polaritonic wave signal to be used for manual detection of polariton wavelength (note a non-uniform separation between peaks). (e) sSNOM aligned map as obtained by numerical transformation of (d) to keep edge along vertical direction.

In contrast to “ordinary” wave-peak counting approach, which is ambigous, we apply fully numerical analysis via the eikonal wave. We derive the wavevector of polariton by detecting the polaritonic eikonal phase derivative, $-i\partial\phi/\partial x$, which we compute moving across the edge boundary. The result is shown in Figure S12a. Importantly, the wavevector in the eikonal model is not global but rather a local quantity (depends on the wave location). While this gives an advantage to materials characterization, it also requires a careful analysis. Specifically, in case of polariton waves reflected from the edge, the propagation constant has an opposite sign to the left and right side of the interface. As a result, the phase of the wave incident from the left should be decreasing (with negative derivative), and then, after edge point, increasing (with positive derivative). It is well known that the sSNOM signal is coming already pre-processed from the Neaspec software. As one can see in Figure S12e, the sign of the phase is exactly opposite, which is a typical artifact of the harmonics’ demodulation. Nevertheless, by knowing the physics of the wave scattering process, one can reproduce the right direction of wave propagation. In our case, the trajectory in the Argand space starts from top (near smaller black fitting circle) and continues downwards (to blue circle). The points in this Argand plot are from the whole map shown in Figure S12d and clearly form two “kissing” arcs for waves reflected off the edge. The central positions of the fitting circles are not the same, which allows one to determine the shift of the sSNOM background signal when moving off the bottom-hBN layer.

Since not only the phase derivative changes the sign but also the background signal (subtracted center position of the fitting circle) is different on two sides of the edge, the curves like in the Figure S12a are different for fitting on either side of the interface (here we only get the values on the right). However, one can see a good convergence of the wavevector to the value derived from the manual wavelength count (depicted by the blue horizontal gridline).


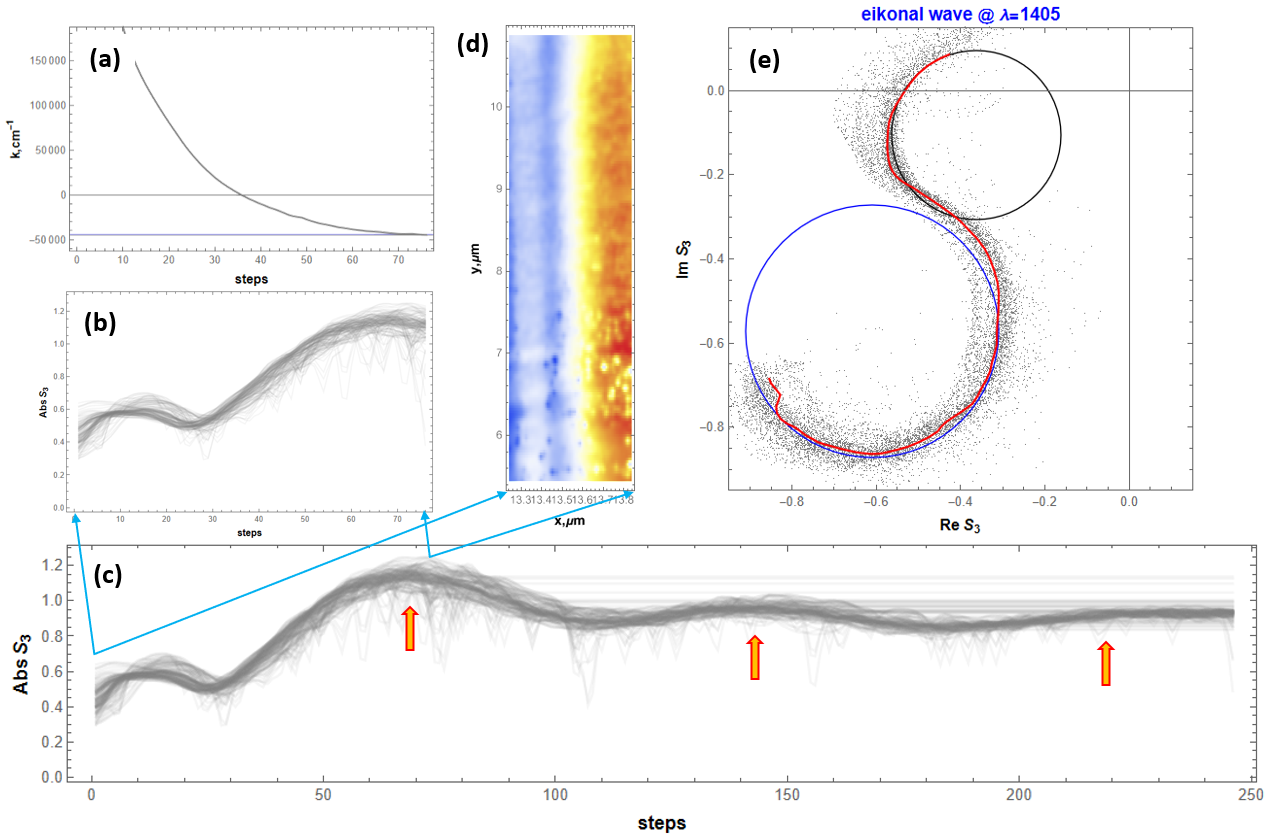


**Figure S12.** Eikonal wave analysis of a single polaritonic sSNOM map. (a) Convergence of the polaritonic phase derivative ($-i\partial\phi/\partial x$), equal to the wavevector of polariton to the right of the edge, to the “ordinary” value (blue gridline) obtained from the manual wavelength detection (cf. the distance between the arrows in panel (c)). Partial (b) and full (c) polaritonic wave profile to the right of the edge. (d) sSNOM map showing actual data, used to plot profiles in (b). (e) An Argand plot of the whole map in (d). Red curve is an average phase trajectory of a polariton wave, used to get the wavevector in (a); black and blue fitting circles allow to find and subtract the value of sSNOM background, different on two sides of the edge.

The Argand plot in Figure S12e shows a limited part of the wave pattern (note that neither blue nor black arc has more than 180^o^ arc length). Actual area taken for analysis (a fraction of the whole map in Figure S11d) is shown in Figure S12d. Corresponding wave profiles are plotted in Figure S12b. Note that after data alignment, all profile curves collapse. For the purpose of comparison, we present also much longer profile curves in the panel (c) – that amount of data would be needed to manually determine the “ordinary” wavelength, using the distance between the maxima indicated by arrows. As one can judge the manual wavelength requires averaging over the large distance and is not free from ambiguities: below we will use manual wavevectors for comparison with the eikonal results (more than one fit is possible, thus for some maps, we will present several values of a manual wavevector).

For theoretical analysis of the polaritons in the sandwich material we used a classical model of light reflecion from a stratified medium. It is well known that the polaritonic modes of such a medium correspond to the poles of the total reflection coefficient, R, and can be visualized when plotting R vs. the frequency and wavevector of the polariton. Each layer is characterized by a frequency dependent dielectric tensor^[[1]](#endnote-1)^ (for uniaxial hBN), or a single dielectric function^[[2]](#endnote-2)^ (for isotropic amorphous SiO_2_), or a single value of the dielectric constant^[[3]](#endnote-3)^ eps=11.7 (for Si, which is known to have negligible dispersion in the mid-IR region), or a two-dimensional optical conductivity^[[4]](#endnote-4)^ (for room-temperature Dirac model for graphene, with the dependence on the doping level). Although the parameterization of the model does not change the result qualitatively, it may make the dispersion curves to slightly deviate from measured values.

Figure S13 presents spectral data used in this section: panel (a) shows Re and (negligible) Im part of the dielectric function used for amorphous SiO_2_ layer; panel (b) shows two-dimensional optical conductivity of a monolayer graphene for n_G_=2x10^12^ cm^-2^ doping level (best fit); since the 2d-conductivity has the units of velocity it was divided by the speed of light for making a unitless quantity; panel (c) shows Re and Im parts of both components of dielectric tensor of hBN (ordinary/in-plane, and extraordinary/out-of-plane).

We construct the propagators for electromagnetic waves in each layer as:

$$k_{n}=\sqrt{\varepsilon_{n} \beta^{2} - q^{2}}$$

for the dielectric functions of isotropic media: n = 2 [Si], or n = 1 [SiO2], or n = 0 [air ($\varepsilon=1$)], where $\beta=\frac{\omega}{c}$, here c is the speed of light, ω is the frequency, q is the polariton wavevector (in plane).


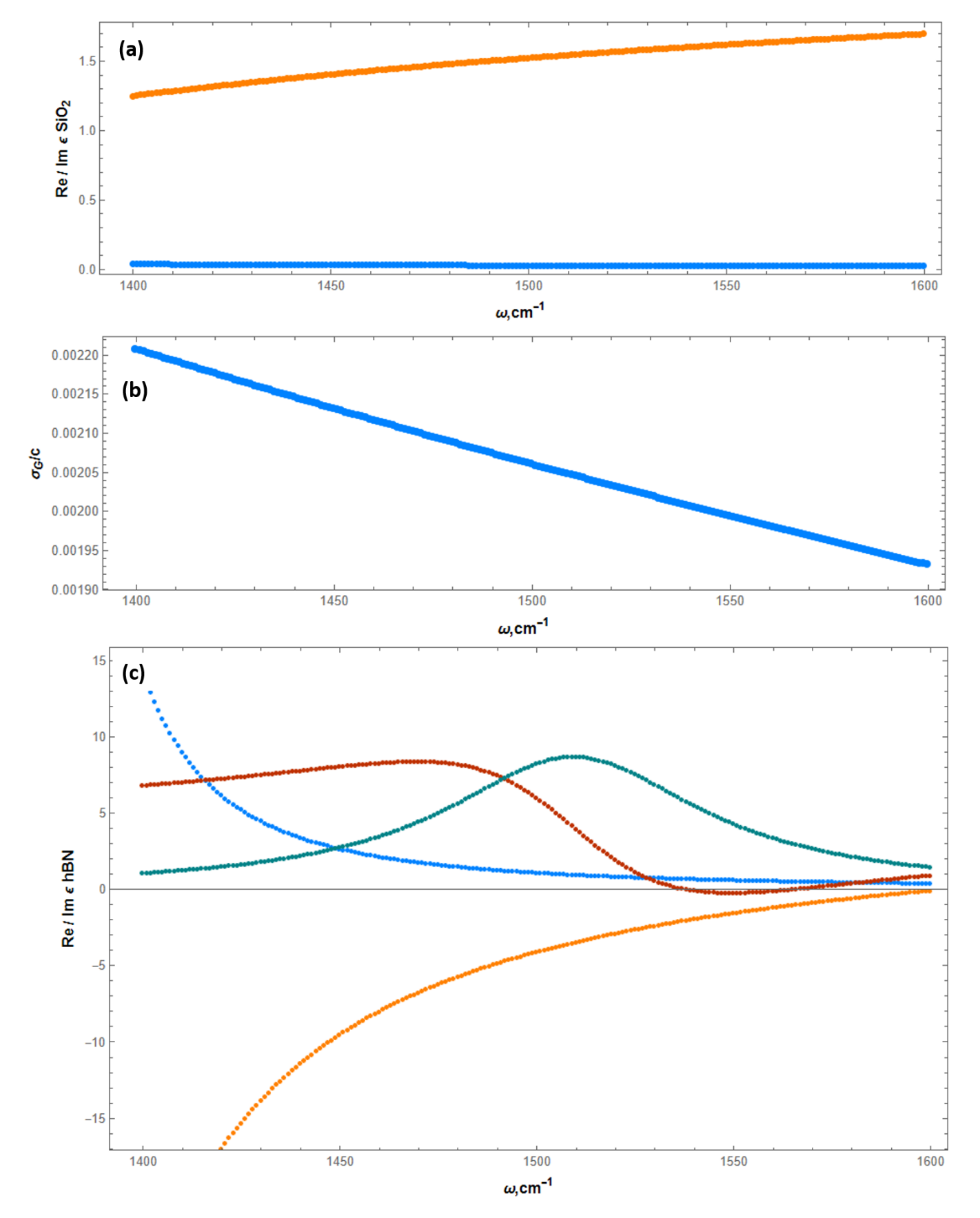


**Figure S13.** The dielectric function parameters used in this section: (a) Re (orange) and Im (blue) parts of the dielectric function for amorphous SiO_2_ [2]; (b) two-dimensional optical conductivity of monolayer graphene [4], divided by the speed of light, for doping level: n_G_=2x10^12^ cm^-2^; (c) Re (orange) and Im (blue) parts of ordinary/in-plane components of the dielectric tensor of hBN; Re (maroon) and Im (emerald) parts of extraordinary/out-of-plane components [1]. All functions are shown in the spectral range 1400-1600 cm^-1^.

The propagator for an isotropic medium (hBN) reads as:

$$k_{hBN}=\sqrt{\varepsilon_{hBN}^{e} (\beta^{2} - q^{2}/\varepsilon_{hBN}^{o})}$$

where the in-plane and out-of-plane components of the dielectric tensor of n = hBN are given by $\varepsilon_{hBN}^{o}$, and $\varepsilon_{hBN}^{e}$. Finally, we introduce the parameter for MLG with the units of propagator:

$$Q=\frac{i \omega}{2\pi\sigma}$$

where $\sigma$ is the optical conductivity:

$$\sigma=i\frac{e^{2} v_{F}}{\pi\hbar\omega}\sqrt{{\pi n}_{G}}$$

where $v_{F}$ ~10^6^ cm/s is the Fermi velocity;

With these parameters, we define the reflection coefficients between layers n and m as:

$$r_{m}^{n}=\frac{k_{n}/\varepsilon_{n} -k_{m}/\varepsilon_{m}}{k_{n}/\varepsilon_{n}+k_{m}/\varepsilon_{m}}$$

where for the anisotropic medium one substitutes $\varepsilon_{hBN}^{e}$ for the dielectric function. While for graphene monolayer we need:

$$r_{G}=\frac{q}{q - Q}$$

The Fresnel reflection coefficient of the whole sandwich slab t-hBN/MLG/b-hBN/SiO_2_/Si is analytically calculated using the transmission matrix approach. Each layer (except for MLG) contributes a factor T`.G.T, where G is the free propagation matrix, and T-matrices (interface matrices) are written via the $r_{m}^{n}$ coeffcicients. The total transmission matrix reads as:

T =$\left[ \begin{matrix} \frac{1}{1+r_{3}^{2}} & \frac{r_{3}^{2}}{1+r_{3}^{2}} \\ \frac{r_{3}^{2}}{1+r_{3}^{2}} & \frac{1}{1+r_{3}^{2}} \end{matrix} \right]$.$\left[ \begin{matrix} e^{i\Delta} & 0 \\ 0 & e^{-i\Delta} \end{matrix} \right].\left[ \begin{matrix} \frac{1}{1+r_{2}^{1}} & \frac{r_{2}^{1}}{1+r_{2}^{1}} \\ \frac{r_{2}^{1}}{1+r_{2}^{1}} & \frac{1}{1+r_{2}^{1}} \end{matrix} \right].$ $\left[ \begin{matrix} e^{i\Delta_{b}} & 0 \\ 0 & e^{-i\Delta_{b}} \end{matrix} \right].\left[ \begin{matrix} \frac{1}{1-r_{1}^{0}} & \frac{-r_{1}^{0}}{1-r_{1}^{0}} \\ \frac{-r_{1}^{0}}{1-r_{1}^{0}} & \frac{1}{1-r_{1}^{0}} \end{matrix} \right].\left[ \begin{matrix} \frac{1-2r_{G}}{1-r_{G}} & \frac{r_{G}}{1-r_{G}} \\ \frac{-r_{G}}{1-r_{G}} & \frac{1}{1-r_{G}} \end{matrix} \right].\left[ \begin{matrix} \frac{1}{1+r_{1}^{0}} & \frac{r_{1}^{0}}{1+r_{1}^{0}} \\ \frac{r_{1}^{0}}{1+r_{1}^{0}} & \frac{1}{1+r_{1}^{0}} \end{matrix} \right].\left[ \begin{matrix} e^{i\Delta_{t}} & 0 \\ 0 & e^{-i\Delta_{t}} \end{matrix} \right].\left[ \begin{matrix} \frac{1}{1-r_{1}^{0}} & \frac{-r_{1}^{0}}{1-r_{1}^{0}} \\ \frac{-r_{1}^{0}}{1-r_{1}^{0}} & \frac{1}{1-r_{1}^{0}} \end{matrix} \right]$

There are 4 fitting parameters we chose to be close to experimentally measured physical ones: thickness of t-hBN and b-hBN layers, taken as 5 nm and 340 nm; thickness of SiO_2_ layer of 285 nm (nominal); doping level of MLG: n_G_=2x10^12^ cm^-2^. As mentioned earlier, the results are not too sensitive to the choice of these parameters. The Fresnel coefficient is deduced from matrix elements of the total transmission matrix as:

$$R=\frac{{-T}_{12}}{T_{22}}$$

The density map of the imaginary part of Fresnel coefficient R shows all polaritons modes in the slab. While low-q (long-wavelength) modes have complicated structure (not to be analyzed here), the modes in the region of interest (20,000-120,000 cm^-1^) are coming mostly from the waves bound to the hBN due to the spectral region of interest (1400-1600 cm^-1^) is within the Reststrahlen band of hBN (see Figure S13c). One of these modes is clearly visible as a C-shape curve in Figure 14. All red symbol data points, derived from the raw sSNOM maps using the eikonal model, above ~1420 cm^-1^ fall on this curve (a few points at the bottom of dispersion curve show coupling of this mode to a longer wavelength mode below it). Notably, the manual fit of the polariton wavelength (points of other color without error bars) show similar trends, though have a significant scatter and contain several outliers.


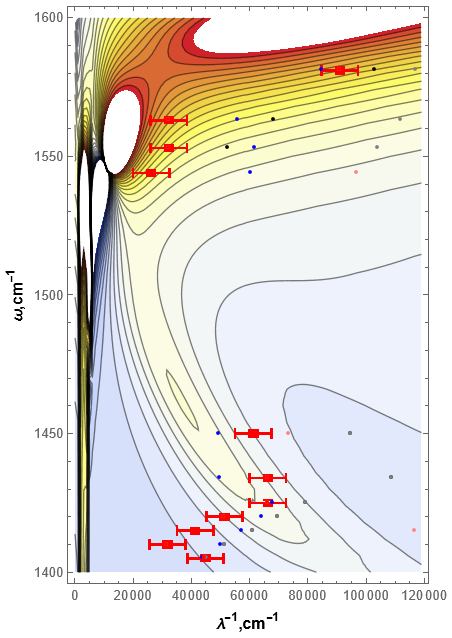


**Figure S14.** The comparison of eikonal model for sSNOM-based polariton dispersion curve (red symbols with error bars), with the manual wavelength fitting (all other points), and the theoretical prediction (density plot) Im(R) for the t-hBN/MLG/b-hBN/SiO_2_/Si slab with the parameters described in the text.

In conclusion, here we demostrate that the eikonal method allows to determine polariton wavevectors that have quantitive agreement with the classical theory for multilayer slab polaritons.

**S9. Polariton dispersion in non-structured SiC/2D-Ag/EG films**

Figure S15 and S17 show the sSNOM maps of non-structured SiC/2D-Ag/EG films, chosen from the hyperspectral series SH1 (76 single laser frequency maps) and SH2 (62 single laser frequency maps). We stress that since the material morphology is highly non-uniform – it demonstrates multiple terrace steps, other layer non-uniformities, line and point defects – the propagation of free polariton waves is highly impeded by these morphological features that appeared at the distances much shorter than the polariton wavelength. As a result, the polaritons are frequently scattered and show a complex diffraction pattern. We provide a few typical maps in Figure S15 and S18. As one can see, no clear wave pattern can be traced in these maps, completely prohibiting simple real-space wavelength analysis (counting wavelength distance from the maps).

Nevertheless, using Fourier transform of the maps (with Gaussian filtering of the data to remove high-frequency components which could produce a significant noise) one can trace several spots in the 2D Fourier space that correspond to the reflexes of the scattered polariton waves. For complex film morphology, multiple scattering events are frequent and, therefore, in the Fourier space one can detect typically multiple harmonics of the wavevectors, that show as a series of peaks at commensurate values along the same direction in the reciprocal space.


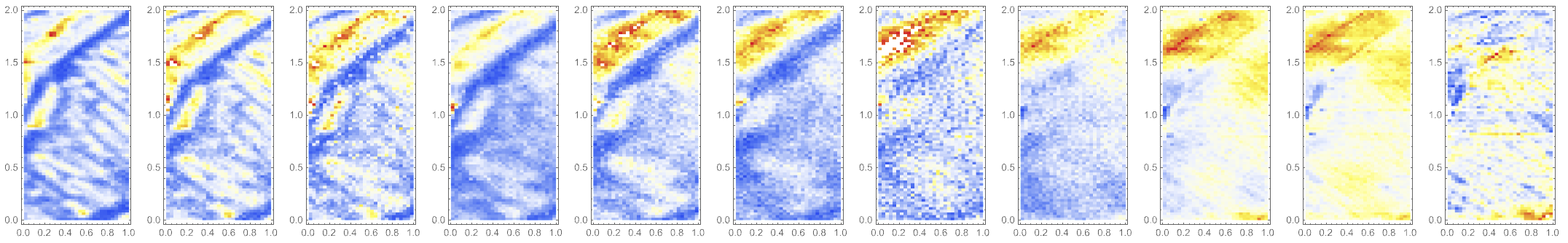


**Figure S15.** Typical raw sSNOM O3A maps of the hyperspectral series for sample SH1. Each map is taken over the same area 1um x 2um and contains 120,000 pixels (this figure does not show full resolution of the map). Total series covers the spectral range 925-1000 cm^-1^, with the step of 1 cm^-1^ (only few maps are shown here).

Figure S16 shows a typical 2D Fourier map of one of the images in Figure S15 – a large central peak of zero frequency reflects long-wavelength fluctuations over the map area, seen in real-space maps. The red line shows direction in the reciprocal space which we chose to plot the polariton dispersion. Multiple peaks along this line correspond to several harmonics.

Figure S17 (left) shows a hyperspectral representation of the Fourier transform of the raw sSNOM map of O3A channel, which is a single wavevector/single laser frequency component for the near-field amplitude (To process the 2D Fourier data a Gaussian filter has been applied to remove high-frequency noise; the data was then interpolated onto a uniform grid and re-sampled along a specific direction in the reciprocal space; then, the FT data for each laser wavelength of the measured hyperspectral series was combined into the density map).


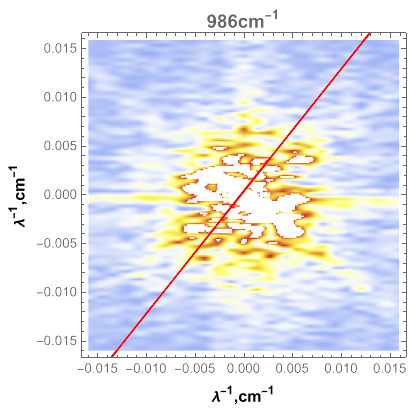


**Figure S16.** 2D Fourier map of the sSNOM O3A map at 951 cm^-1^, shown in Fig. S15 (#9). Red line shows the direction for taking spectral dispersion.


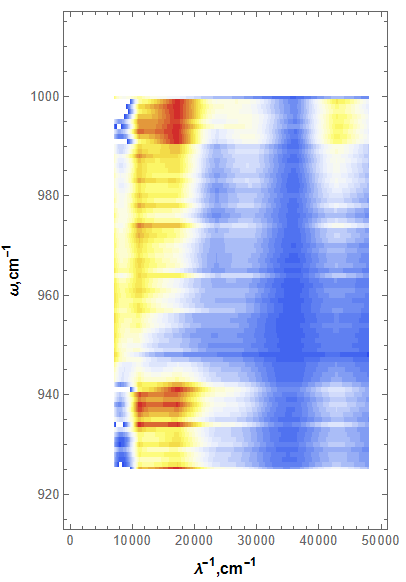

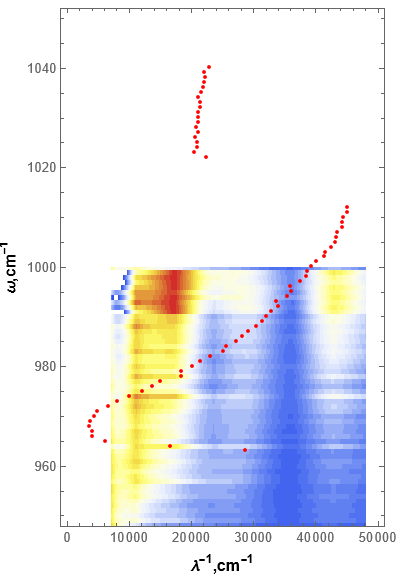


**Figure S17.** Hyperspectral sSNOM dispersion map for non-structured SiC/2D-Ag/EG film (no plasmonic dots fabricated) (hyperspectral series for sample SH1). (left) Actual measured data; (right) same map, rescaled to be overlaid with the dispersion curve from Fig. 4d (in main text).

One can clearly trace several features of the dispersion map: major polariton dispersion branch with a steep slope (for inverse wavelength values between 10,000 and 20,000 cm^-1^); discontinuity of the dispersion of this branch near 945 cm^-1^ frequency; multiple harmonics of the polaritons (due to multiple reflections) that show up as nearly parallel lines/commensurate wavevectors; notably, the amplitude of the higher harmonics decays below certain frequency.

Interestingly, Figure S17 (right) shows the hyperspectral series SH1 compared with the dispersion curve of a pQD sample from Figure 4d (main text). It can be traced that the upper branch of the pQD polariton (red dots) dispersion follows the line of “bulk” polariton – unfortunately, in this SH1 series, the spectral data for bulk sample cannot be explicitly traced in the region of interest. This problem has been resolved in 2nd hyperspectral series below. The lower branch of pQD polariton, in its upper part above 1000 cm^-1^ frequency, also follows the high-k harmonic of bulk polariton (around 45,000 cm^-1^).

There is a visible feature near 965 cm^-1^ in pQD dispersion (a broad S-shape/inverse dispersion part of the pQD-SPP line) which is in accordance with the narrow, though resolvable feature of the bulk-SPP. Not all the features of the plasmonic confined sample and the bulk one are the same: for example, a similar narrow feature of the bulk-SPP near 974 cm^-1^ has no corresponding signature for pQD.

Finally, the discontinuity feature of pQD polariton: the spectral gap between upper branch and lower branch has a correspondence with the amplitude of the high-k harmonics of the bulk-SPP: the high wavevector harmonics have negligible amplitude in the region below the pQD lower branch curve. All these observations are more clearly evidenced in next example SH2.

Figure S18 shows a few near-field maps from SH2 series, taken in the laser frequency range 916-1039 and 1084-1105 cm^-1^, with the step of 3 cm^-1^ (the gap is due to capability of the MIR laser we used). This is a similar non-structured SiC/2D-Ag/EG film material, though we chose a different sample to demonstrate reproducibility of the results.


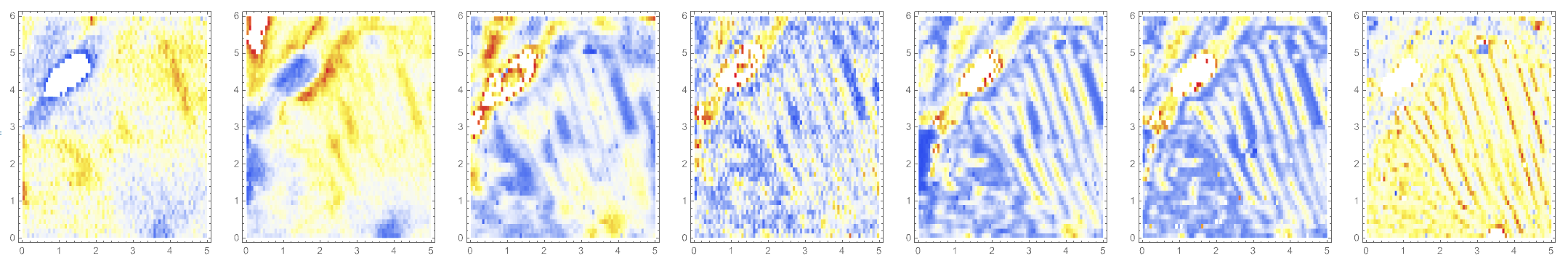


**Figure S18.** Typical raw sSNOM O3A maps of the hyperspectral series for sample SH1. Each map is taken over the same area 5um x 6um and contains 192,000 pixels (this figure does not show full resolution of the map). Total series covers the spectral range 916-1105 cm^-1^, with the step of 3 cm^-1^ (only few maps are shown here).

In this SH2 series one can clearly see that the pQD upper branch follows one of the bulk SPP high-k harmonic dispersion. Indeed, the pQD lower branch curve closely follows the boundary of the (upper) bulk SPP region where the amplitudes of the high-k harmonics are non-vanishing. The bulk SPP dispersion clearly demonstrates spectral features near 1025 cm^-1^, which coincides with the beginning of the upper branch of the pQD polariton, and another one near 965 cm^-1^, where the S-shape of pQD polariton has the minimum wavevector.

Overall, we speculate that close resemblance of the bulk SPP data in the whole studied spectral region of pQD confined polariton dispersion is due to the same physics of MIR polaritonic response of the SiC/2D-Ag/EG material, properly captured by new spectroscopic technique introduced in this paper.


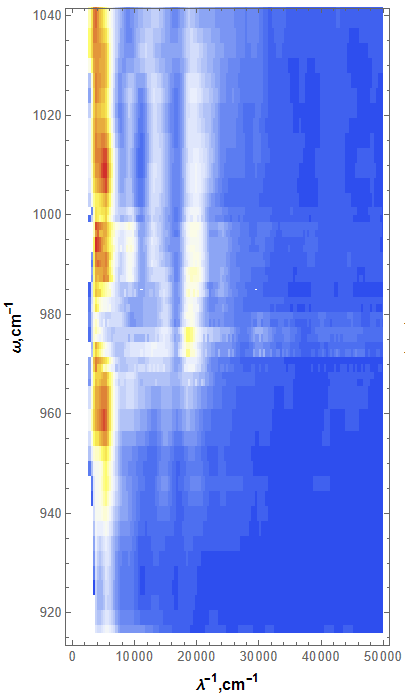

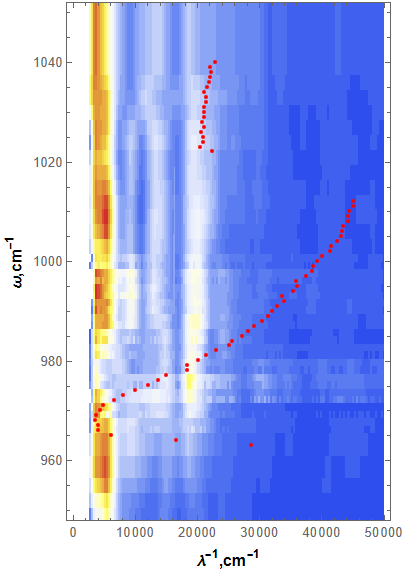


**Figure S19.** Hyperspectral sSNOM dispersion map for non-structured SiC/2D-Ag/EG film (no plasmonic dots fabricated) (hyperspectral series for sample SH2). (left) Actual measured data; (right) same map, rescaled in the same spectral region as in Fig. S17 to be overlaid with the dispersion curve from Fig. 4d (in main text).

**References**

1. Geick R, Perry CH, Rupprecht G. Normal Modes in Hexagonal Boron Nitride. Physical Review. 1966;146(2):543-7. doi: 10.1103/PhysRev.146.543. [↑](#endnote-ref-1)
2. Gervais F, Piriou B. Temperature dependence of transverse and longitudinal optic modes in the α and β-phases of quartz. Physical Review B. 1975;11(10):3944-50. doi: 10.1103/PhysRevB.11.3944. [↑](#endnote-ref-2)
3. Optical Constants of Crystalline and Amorphous Semiconductors. Sadao Adachi. Springer Science+ Business Media, LLC. Originally published by Kluwer Academic Publishers in 1999. [↑](#endnote-ref-3)
4. Woessner, A., Lundeberg, M., Gao, Y. et al. Highly confined low-loss plasmons in graphene–boron nitride heterostructures. Nature Mater 14, 421–425 (2015). https://doi.org/10.1038/nmat4169 [↑](#endnote-ref-4)
